# Recent Developments in Porphyrin-Based Metal–Organic Framework Materials for Water Remediation under Visible-Light Irradiation

**DOI:** 10.3390/ijms25084183

**Published:** 2024-04-10

**Authors:** Nirmal Kumar Shee, Hee-Joon Kim

**Affiliations:** Department of Chemistry and Bioscience, Kumoh National Institute of Technology, Gumi 39177, Republic of Korea; nirmalshee@gmail.com

**Keywords:** porphyrin, metal–organic framework, toxic pollutants, photocatalysis

## Abstract

Access to clean drinking water is a basic requirement, and eliminating pollutants from wastewater is important for saving water ecosystems. The porous structure and surface characteristics of metal–organic frameworks (MOFs) can function as a perfect scaffold for removing toxic compounds from wastewater. Porphyrins are promising building blocks for constructing MOFs. Porphyrin-based metal–organic frameworks (P-MOFs) have been fabricated using porphyrin ligands, metal clusters, or ions. These materials can harvest light from a wide region of the solar spectrum, and their framework morphology and physicochemical properties can be controlled by changing their peripheral subunits or metal ions. These porous crystalline materials have generated interest because of their distinctive characteristics, including large permanent porosity, interesting surface morphology, broad conformational diversity, high photostability, and semiconducting nature. This article discusses the recent progress and usefulness of P-MOFs. The fabrication procedures of P-MOFs are discussed, followed by the adsorptive and photocatalytic removal of contaminants from wastewater. The relationships between the geometries of P-MOFs and their light-harvesting and charge-transfer mechanisms for the photocatalytic degradation of pollutants are highlighted. Finally, some future perspectives and obstacles in the photodegradation usage of P-MOFs are discussed, along with feasible research directions to standardize efficient photocatalysts for improved photodegradation for water treatment.

## 1. Introduction

Environmental pollution has a profound effect on Earth’s biosphere. The rapid growth of industrialization and civilization has caused air and water pollution. Each day, large amounts of hazardous materials, including industrial dyes, pigments, plasticizers, herbicides, pesticides, phenols, biphenyls, amino, and nitro chemicals, are discharged from paper printing, leather, textile, drug, and chemical factories into water ecosystems. These harmful contaminants are not only a major threat to the living world but also deteriorate drinkable water [1,2,3]. At present, more than 50% of the Earth’s population lives in water-scarce regions, and this is predicted to become worse in the coming days. Therefore, providing universal access to safe, drinkable water is a priority of the United Nations. Fresh water is harvested from either groundwater or seawater after desalination. However, the technologies required to perform it efficiently are not affordable worldwide. This has led to growing scientific investigations by environmentalists and researchers to develop strategies for the environmental remediation of wastewater [4,5,6,7,8,9,10,11]. 

Various physicochemical technologies, including precipitation and filtration [12], adsorption [13], electrochemical methods [14], bacterial treatment [15], chemical coagulation [16], and advanced oxidation processes (AOPs) [17], have been developed to remove hazardous chemicals from wastewater. AOPs are the most suitable methods for water remediation because of their low cost, simple procedure, and impressive performance in the decomposition of contaminants into less toxic H_2_O and CO_2_ without generating any other contaminants. In AOPs, an appropriate catalyst absorbs light and produces reactive oxygen species (ROS) in situ, accelerating the decomposition of hazardous contaminants in wastewater. In general, the process of visible-light photocatalysis has received substantial recognition because it can provide ecological and economic benefits. The mechanism is simple and consists of two steps. In step one, light harvesting is followed by exciton diffusion. In step two, charge separation is followed by carrier transport. Consequently, light absorption followed by sequential electron transfer plays a crucial role in a catalyst attaining the optimum solar energy harvesting capability [18,19,20,21,22,23].

In photofunctional materials, nanoscale architectures usually exhibit unique optoelectronic properties depending on their dimensions. Therefore, the construction and design of nanoscale architectures are crucial for the construction of photocatalyst systems with specific properties and functions. Porous materials can absorb pollutants from aqueous solutions and effectively separate them from the wastewater. Considering the superiority of photodegradation techniques, various inorganic and organic materials have emerged as new building blocks for fabricating photocatalysis schemes. Among various inorganic materials, metal oxide nanoparticles (MNPs) (TiO_2_ [24] and ZnO [25]), graphitic carbon nitride (g-C_3_N_4_) [26], and bismuth-based catalysts [27] have been used as photocatalysts for pollutant degradation. However, their application is greatly restricted by their low light-harvesting ability under ultraviolet light irradiation, limited adsorption capacity, fast recombination, and low cycling stability. Moreover, a large amount of the catalyst is essential for initiating the photocatalytic process and achieving effective photodegradation rates. Therefore, it is important to fabricate materials that can overcome these limitations [28].

Metal–organic frameworks (MOFs) have been used as potential candidates owing to their extensive applications in gas storage and separation [29], catalysis [30], biomedicine [31], drug delivery [32], sensing [33], and energy storage systems [34]. Generally, MOFs are complexes comprising organic linkers coordinated with metal ions or clusters to fabricate one-, two-, and three-dimensional frameworks. Therefore, MOFs present a distinctive platform to address the above drawbacks owing to their high permanent porosity and synthetic utility, which allows researchers to control their structures and pores. The advantages of these porous compounds are mostly due to their coordination architectures, functional active sites, and permanent void spaces inside the pores. Therefore, it is important to select suitable metal ions and organic building blocks with appropriate coordination frameworks to control the conformational structures of these porous compounds [35,36,37].

Among the different building block units of organic functional connectors that have been utilized in fabricating MOFs, porphyrin-based metal–organic frameworks (P-MOFs) have gained recognition owing to their tunable molecular geometries and excellent optoelectronic properties [38,39,40,41]. Several P-MOFs have been widely considered because of their extensive use in gas storage and dissociation [42], metal ion detection [43], hydrogen production [44], biomedical applications [45], solar energy conversion [46], CO_2_ reduction [47], cancer treatment [48], fuel cells [49], heterogeneous catalysis [50], and water purification [51]. Porphyrin compounds are extensively found in nature, for example, hemoglobin in animal blood for transporting O_2_, catalase for the degradation of H_2_O_2_, chlorophylls in autotrophs for photosynthesis, vitamin B_12_ for cell metabolism, and cytochromes for several oxidative processes [40,52,53]. Porphyrins are a group of *N*-heterocycle compounds that have (4n + 2) π electrons (26 π, n = 2) and are associated with the linkage of the α-carbon atoms of four pyrrole secondary units via a methine bridge. Highly conjugated free porphyrins or metalloporphyrins possess chromophores, and both absorb visible light. The typical peaks of porphyrins in the visible region are a Soret band with a spectral range of 400–450 nm and four weak Q bands with a spectral range of 500–710 nm. The Soret band (strong absorption) and Q bands (weak absorption) are associated with the π → π* electron transfer within unsaturated porphyrin moieties. However, these optical characteristics can be altered synthetically by changing the central metal atom and peripheral side group functionality [54,55,56,57,58]. In solution, their fixed geometrical skeletons and inherent aromatic electronic features lead to their self-aggregation. Different non-covalent intermolecular interactions, such as electrostatic interactions, hydrogen bonding interactions, π-π stacking interactions, metal-ligand coordination interactions, and hydrophilic and hydrophobic interactions, are responsible for the self-aggregation of porphyrin compounds [59,60,61,62,63,64].

P-MOFs are commonly fabricated via supramolecular interactions, including peripheral coordinative bonds with the metals of neighboring metalloporphyrins, aggregated via H-bonding interactions, or external metal ions or clusters [65,66]. The axial coordination of metalloporphyrins can also produce stable and rigid molecular structures [67,68,69,70]. Moreover, porphyrins allow the modification of the inner cavity of metal–organic frameworks through unsaturated metal coordination and can bind metal ions or clusters to serve as active sites for catalytic applications [71,72]. The combination of permanent porosity and the catalytic properties of P-MOFs encouraged us to investigate their usefulness as multifunctional adsorbents and catalysts. The formation of these materials not only increased their light-harvesting capacity but also enhanced their recyclability for further use. Additionally, the fixed structural network prevents hydrolysis and increases photostability. Therefore, P-MOFs are appropriate platforms for organizing porphyrin molecules into mesoporous architectures and controlling their surface area, morphology, and photocatalytic properties [73,74]. Therefore, the fabrication of P-MOFs from porphyrin ligands and metal ions or connectors not only modifies the structural topologies but also generates high conformational changes, such as high chemical stability, large permanent porosity, excellent surface morphology, a high degree of active sites, and excellent catalytic photodegradation ability against toxic contaminants under visible-light irradiation. The effective light-harvesting ability in the visible-light region, significant electron transfer properties, large number of active sites, and enormous stability of P-MOFs make them unique compared to other visible-light photocatalysts. Therefore, P-MOFs constructed by incorporating porphyrin molecules with metal ions or clusters are multifunctional carriers with outstanding catalytic photodegradation activity and desired functionality.

Recently, various reviews have reported the progress achieved in the development of porphyrin-based metal–organic frameworks and their applications in energy-related fields [75,76,77,78,79]. However, an extensive understanding of the relationship between porphyrin-based metal–organic frameworks and their photocatalytic performances in water treatment is lacking. The aim of this review is to discuss the current advancements in P-MOFs, their utilization in environmental remediation, and the future perspectives of P-MOFs as potential photocatalysts. This review will inspire material chemistry researchers to design more efficient P-MOFs for water remediation and energy-related applications.

## 2. Strategies for Construction of P-MOFs

Depending on the nature of the bonding between the porphyrin ligands and metal ions or clusters, P-MOFs can be classified into two categories: porphyrinic MOFs and porphyrin@MOFs. In porphyrinic MOFs, porphyrin acts as an organic linker and chelates metal ions or clusters to form P-MOFs. The structure of these MOFs is formed by intermolecular interactions, such as covalent bonds, hydrogen bonding, π-π stacking interactions, van der Waals forces, and coordination bonds. In contrast, the one-pot synthesis of porphyrin ligands with MOF precursors induces the formation of porphyrin@MOFs. Porphyrin ligands are incorporated inside the cavities of the MOF precursors and then coordinated with metal ions or ligands via host–guest interactions. Several approaches have been used to construct P-MOFs. Various reliable processes, such as hydrothermal, solvothermal, microwave-assisted, sonochemical, mechanochemical, electrochemical, reverse microemulsion, slow evaporation, and vapor diffusion processes, have been used for the formation of MOFs. This section is divided into three parts: (1) P-MOFs derived from 5,10,15,20-tetrakis(4-pyridyl)porphyrin (H_2_TPyP) and related pyridyl-based ligands, (2) P-MOFs associated with 5,10,15,20-tetrakis(4-carboxyphenyl) porphyrin (H_2_TCPP) and other carboxyphenyl-related ligands, and (3) P-MOFs constructed from other porphyrin-based ligands.

### 2.1. Pyridyl-Based Porphyrinic MOFs

Free H_2_TPyP and its different metallated derivatives (MTPyPs) are versatile elementary units used for the synthesis of P-MOFs. H_2_TPyPs (or MTPyP) act as tetratopic planar μ^4^-bridging donor ligands with four peripheral pyridyl groups. Peripheral pyridine linkers are oriented in a tetragonal conformation and assembled using external mononuclear metal clusters or ions to fabricate square planar frameworks of infinite parallel sheets.

**Figure 1:**
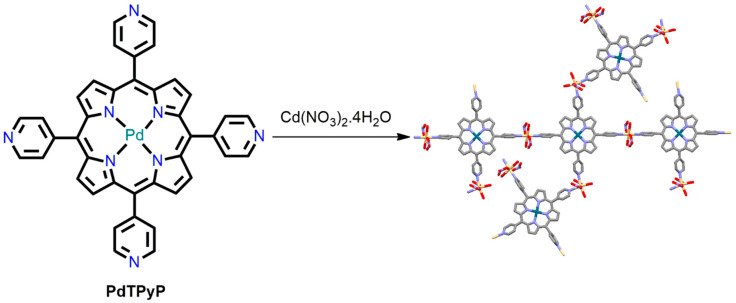
A view of the PdTPyP and the linked Cd^II^ centers in (PdTPyP∙2Cd(NO_3_)_2_∙hydrate). Adapted from Ref. [80].

In 1991, Robson et al. constructed the first porphyrin-based three-dimensional (3D) coordination polymers using a one-step solvothermal reaction of [5,10,15,20-tetrakis(4-pyridyl)porphyrinato]palladium(II) (PdTPyP) ligands with Cd^2+^ ions [80]. In a typical reaction, PdTPyP is suspended in cadmium nitrate in a mixed solvent (methanol and water) and refluxed for 24 h. Dark-red crystals are obtained after the evaporation of the solvent at room temperature. Here, the PdTPyP unit serves as a planar, four-connected node, and the Cd(II) ion acts as a connector. The complex shows a new topology in which four pyridyl units of the PdTPyP ligands are interconnected as an infinite 3D network through coordination to the octahedral Cd^2+^ ion. Each Cd(II) ion forms coordinate bonds with two pyridyl groups, two H_2_O molecules, and two monodentate NO_3_^−^ ions. Cd(II) ions are assembled with square planar PdTPyP to form 1D chains that pass over each other to produce a 3D network with a large pore volume (Figure 1).

**Figure 2:**
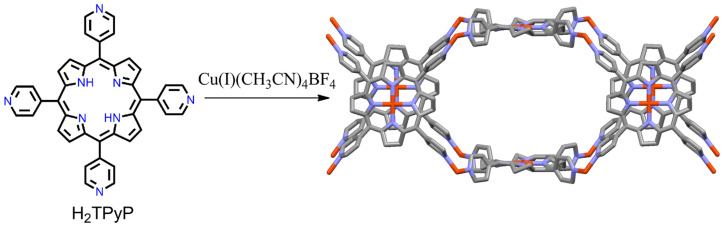
A schematic of the tetragonal unit of {[Cu(II)(TPyP)Cu(I)]_n_}^n+^ network. Reproduced from Ref. [81].

The same group also reported robust coordination network structures containing both Cu(I) ions and CuTPyP ([5,10,15,20-tetrakis(4-pyridyl)porphyrinato]copper(II)) building block units [81]. In a typical procedure, the reaction of Cu(I)(CH_3_CN)_4_BF_4_ with H_2_TPyP in acetonitrile/nitrobenzene under a nitrogen atmosphere, followed by slow evaporation, leads to the formation of dark ruby-red crystal structures with a 3D PTS net topology by coordination with metalloporphyrin building blocks. The combination of the tetrahedral Cu(I) connector and the square-planar geometry of CuTPyP results in the formation of effective nitrogen–Cu bonds within the layered coordination frameworks. Two adjacent tetragonal units of {[Cu(II)(TPyP)Cu(I)]_n_}^n+^ interpenetrate each other, guiding the formation of a 3D PTS network (Figure 2). This tetragonal framework exhibits large channels held by counter-anions and disordered solvent molecules with high thermal stability (up to 300 °C). After removing the solvent from the crystal cavity, the crystalline materials are converted into an amorphous powder.

**Figure 3:**
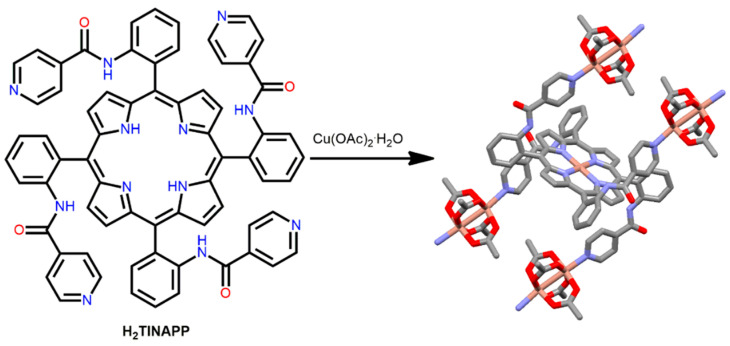
Structure of the 1D coordination framework constructed by the Cu(OAc)_2_·2H_2_O with H_2_TINAPP in *i*PrOH/CHCl_3_. Adapted from Ref. [82].

Kyritsakas et al. reported 1D and 2D coordination frameworks arising from the reaction of Cu(OAc)_2_·2H_2_O and 5,10,15,20-tetrakis(isonicotinoylamidophenyl)porphyrin (H_2_TINAP) [82]. Solvent systems play a key role in the self-assembly of 1D or 2D coordination networks during crystallization. The 1D stair-type framework was fabricated using an *i*PrOH/CHCl_3_ solvent system. In this framework, the Cu-porphyrin moieties are connected through all four isonicotinoyl units of the four Cu_2_(CH_3_COO)_4_ dimers. The pyridine groups are positioned parallel to each other on the same face as one with the Cu-porphyrin ring. The 2D framework was obtained from a 1,2-dichlorobenzene/EtOH solvent system. In the 2D framework, the Cu-porphyrin ligands are connected via the isonicotinoyl groups of the four Cu_2_(CH_3_COO)_4_ dimers. Of the two pyridine rings, one is strongly tilted toward the Cu-porphyrin plane, while the other is perpendicular. H_2_TINAP has four atropisomers owing to the presence of a bulky isonicotinoyl group. The two obtained frameworks can be regarded as structural or supramolecular isomers (Figure 3). 

**Figure 4:**
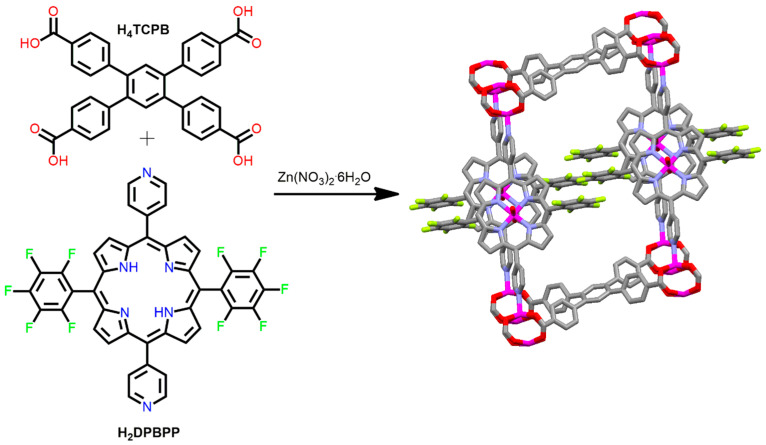
Topology of Zn-MOF constructed from Zn(NO_3_)_2_·6H_2_O, H_4_TCPB, and H_2_DPBPP. Adapted from Ref. [83].

A pillared paddlewheel with stable microporous structures of Zn-MOF was constructed by Nguyen et al. [83]. In a typical process, the reaction of H_4_TCPB (1,2,4,5-tetrakis(4-carboxyphenyl)benzene), H_2_DPBPP (5,15-dipyridyl-10,20-bis(pentafluorophenyl)porphyrin), and Zn(NO_3_)_2_·6H_2_O in DMF (*N*, *N*-dimethylformamide) under refluxing conditions assists in the formation of Zn-MOF. The tetratopic ligand H_4_TCPB acts as a base, and Zn(DPBPP) acts as a pillar to form the paddlewheel Zn-MOF. The tetratopic unit provides framework stability with large channels (gas-accessible surface area of ~500 m^2^/g) (Figure 4).

Moreover, the structural mismatch between the dimensions of the two tetratopic units with strong Zn(II)–carboxylate bonds prevents the available sites of Zn(DPBPP) from functioning as additional nodes. This creates a large, accessible active site that is convenient for the catalytic intermolecular acyl-transfer reaction occurring inside the network.

**Figure 5:**
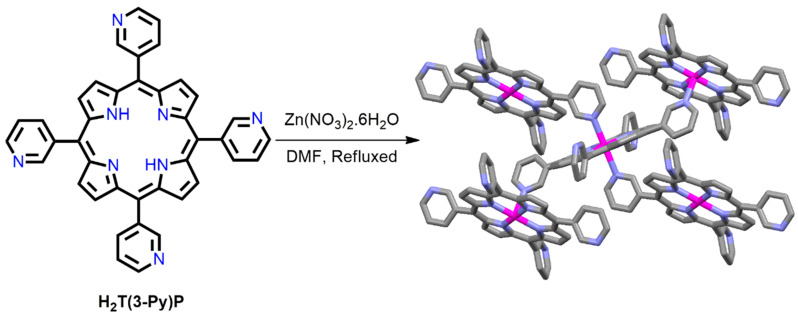
Image shows a 2D coordination network near the Zn^2+^ metal ions in MPF-3. Adapted from Ref. [84].

5,10,15,20-tetra(3- pyridyl)porphyrin, H_2_T(3-Py)P, an analog of H_2_TPyP, is used as an elementary unit for the fabrication of P-MOFs. The orientations of the pyridyl N atoms in H_2_T(3-Py)P are dissimilar from those in H_2_TPyP. Choe et al. reported an adjustable 2D porphyrin framework (MPF-3) obtained from the reaction of Zn(NO_3_)_2_·6H_2_O and H_2_T(3-Py)P in DMF under solvothermal conditions [84]. Only two pyridyl N atoms of each ZnT(3-Py)P porphyrin molecule are axially interlinked via the Zn^2+^ metal ions of the neighboring ZnT(3-Py)P units (Figure 5). The remaining two uncoordinated pyridyl N atoms form the interlocked two-dimensional (2D) layers. The geometry of the MPF-3 framework is very similar to that of the Cairo pentagonal tessellation. The XRD data show that the framework in MPF-3 undergoes a phase change when DMF is removed by heating, and the initial framework is retained when the desolvated phase is submerged in DMF. This confirms the robustness of the coordination network in MPF-3.

**Figure 6:**
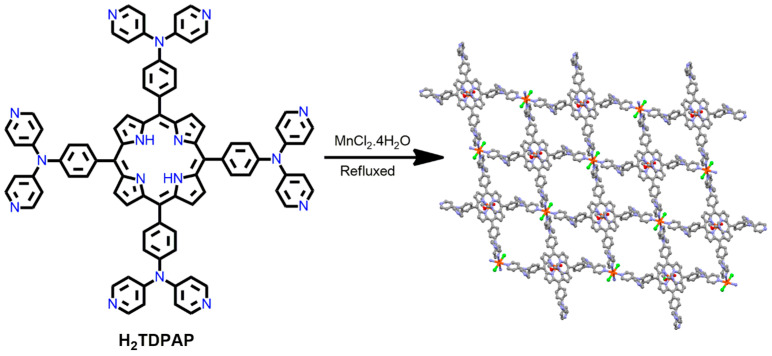
A side view of interpenetrated 2D networks in Mn-MOF. Redesigned from Ref. [85].

Different porous network structures have been obtained from 5,10,15,20-tetrakis(4,4′-dipyridylaminophenylene)porphyrin (H_2_TDPAP) with several metal ions in multiple coordination modes, using its various peripheral pyridines and porphyrin moieties. Xie et al. constructed a novel Mn-MOF from the reaction of MnCl_2_·2H_2_O with H_2_TDPAP in acetonitrile/DMF under refluxing conditions [85]. In metallated porphyrins, the Mn^3+^ ions in the H_2_TDPAP core do not coordinate with the neighboring pyridyl groups of the H_2_TDPAP linkers. This is due to the steric hindrance related to the bulky 4,4′-dpa units in the H_2_TDPAP ligand (Figure 6). Pyridyl rings of adjacent 2D layers are assembled through π···π stacking interactions, which guide the generation of the 3D architecture in the Mn-MOF. Moreover, the 4,4′-dipyridylaminophenylene groups in the H_2_TDPAP ligand rotate around the porphyrin framework, resulting in strong structural flexibility in the Mn-MOF.

**Figure 7:**
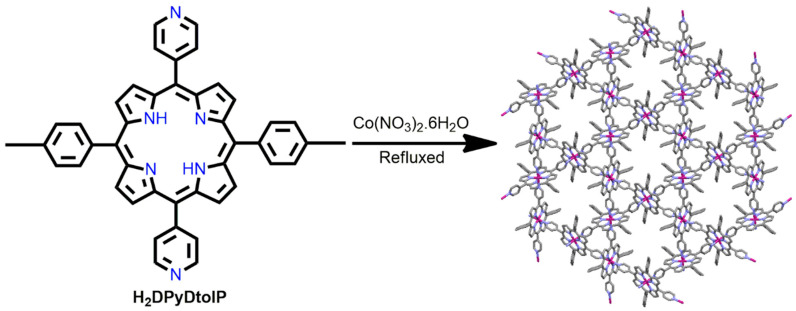
Image shows 3D micropores arranged hexagonally in Co-MOF. Adapted from Ref. [86].

In 2015, Lee et al. demonstrated a stable 3D Co-MOF from the reaction of Co(NO_3_)_2_·6H_2_O with porphyrin derivatives such as H_2_DPyDtolP (5,15-di(4-pyridyl)-10,20-di(4-methylphenyl)porphyrin) under refluxing conditions in DMF [86]. H_2_DPyDtolP is the ditopic ligand. The H_2_DPyDtolP core comprises two 4-tolyl groups (at positions 10, and 20) and two pyridyl groups (at positions 5 and 15). After metallation, the pyridyl groups coordinate and bond with the cobalt ions. The two 4-tolyl moieties create an infinite 3D Co-MOF. The Co-MOFs accommodate micropores that are systematically organized with hexagonal symmetry (Figure 7). Moreover, Co-MOFs exhibit surprisingly high thermal stability at very high temperatures. The Co-MOF retained its crystalline nature even after vacuum drying at 250 °C to remove the solvents. The evacuated Co-MOF absorbed 142.8 cm^3^/g CO_2_ gas at 196 K. CO_2_ and iodine molecules were encapsulated inside the micropore framework of the evacuated Co-MOF at room temperature. A PXRD analysis of the carbon-dioxide-captured Co-MOF revealed linear arrangements of CO_2_ molecules along the 1D framework. In contrast, the PXRD analysis of the iodine-captured Co-MOF confirmed the formation of polyiodide clusters inside the 1D micropore.

**Figure 8:**
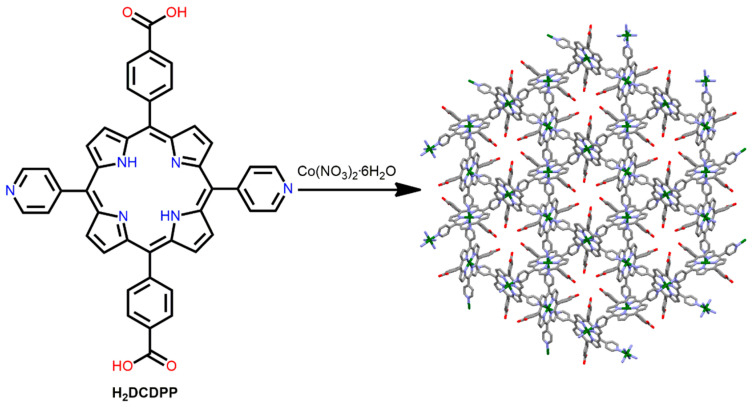
Structure of Co-MOF derived from H_2_DCDPP ligand and Co(NO_3_)_2_·6H_2_O. Adapted from Ref. [87].

Li et al. fabricated a robust Co-MOF by using a mixed bicarboxylate-bipyridyl-substituted porphyrin ligand [87]. The reaction of Co(NO_3_)_2_·6H_2_O with H_2_DCDPP (5,15-di(4-carboxylphenyl)-10,20-di(4-pyridyl)porphyrin) in DMF under refluxing conditions leads to the formation of Co-MOF (Figure 8). The pyridyl N-atoms are coordinated with the Co^II^ ion, and the carboxylic acid groups form H-bonds to create 3D porous networks with large open channels. At 97% relative humidity and 80 °C, the proton conductivity of this framework is 3.9 × 10^−2^ S cm^−1^. This is due to the presence of a large number of non-coordinating carboxyl acid groups positioned in the hydrophobic channel of this Co-MOF. These free carboxylic acid groups can form H-bonds with water molecules, facilitating proton transfer.

**Figure 9:**
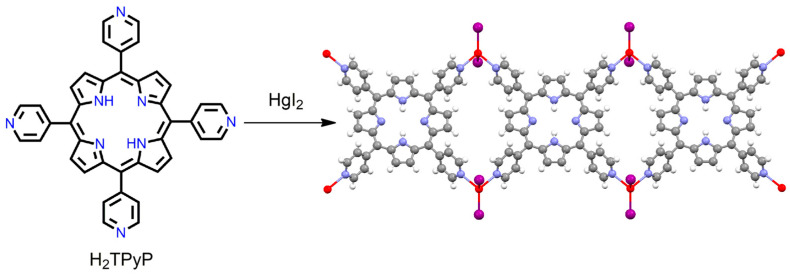
Image shows a 1D coordination polymer in {(HgI_2_)_2_(H_2_TPyP)}_n_·solvated. Adapted from Ref. [88].

Free-base H_2_TPyP can act as elementary units for the formation of MOFs. It provides mostly 1D frameworks with the aid of large connecting metal ions such as Hg^2+^. Rogers et al. reported one-dimensional (1D) frameworks for the reaction of HgI_2_ with free-base H_2_TPyP in mixed solvents [88]. The network structures consist of tetrahedrally coordinated Hg^2+^ cations coordinated with free-base H_2_TPyP ligands to form 1D metallomacrocyclic coordination polymers generated beside the crystallographic *c*-axis (Figure 9). The centroid of the H_2_TPyP moiety lies on a perpendicular mirror plane, and a two-fold rotation axis permits one H_2_TPyP molecule with four symmetry-equivalent pyridine ligands. This type of topology provides hybrid inorganic–organic crystalline materials, in which the polydentate porphyrin ligand is interlinked with the self-assembled porous framework through coordination bonding with the inorganic connector.

**Figure 10:**
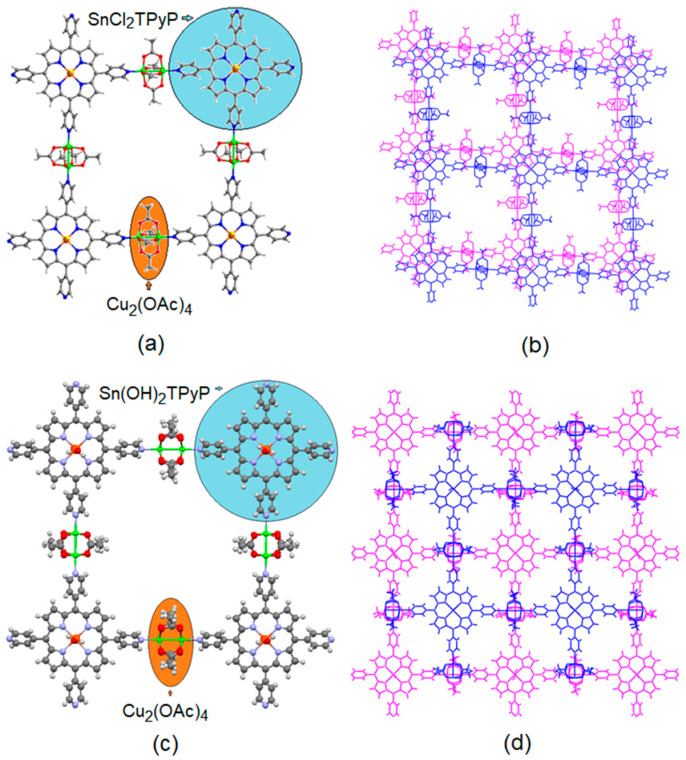
Images show 2D square-grid frameworks in SnTPyP(Cl)_2_·2Cu(OAc)_2_ (**a**,**b**) and SnTPyP(OH)_2_·2Cu(OAc)_2_ (**c**,**d**). Adapted from Ref. [89].

To date, the peripheral coordination of the pyridyl groups of H_2_TPyP or MTPyP with external metal ions or nodes has been used to control the porous architecture of porphyrin-based MOFs. Occasionally, the axial coordination of MTPyP is used to control the porosity of porphyrin-based MOFs. Recently, Kim et al. reported two tin porphyrin-based MOFs in which the axial coordination of tin porphyrin in SnTPyP(X)_2_·4Cu(OAc)_2_·solvated (where X = Cl or OH) controlled the permanent porosity of the MOFs [89] (Figure 10). In a typical procedure, the reaction between Cu(OAc)_2_·H_2_O and SnTPyP(X)_2_ (X = Cl or OH) in a dimethylformamide solvent leads to the formation of two P-MOFs (Figure 10). At 77 K, the Brunauer–Emmett–Teller (BET) surface areas of {[SnTPyP(OH)_2_]⋅[Cu(OAc)_2_]_4_}·solvated and {[SnTPyPCl_2_]⋅[Cu(OAc)_2_]_4_}·solvated were found to be 16.2 m^2^/g and 20.1 m^2^/g, respectively. Trans-axial ligands, such as Cl or OH, coordinated to the Sn(IV)porphyrin center produce unique packing structures with negligible differences in permanent porosity.

### 2.2. Carboxyphenyl-Based Porphyrinic MOFs

**Figure 11:**
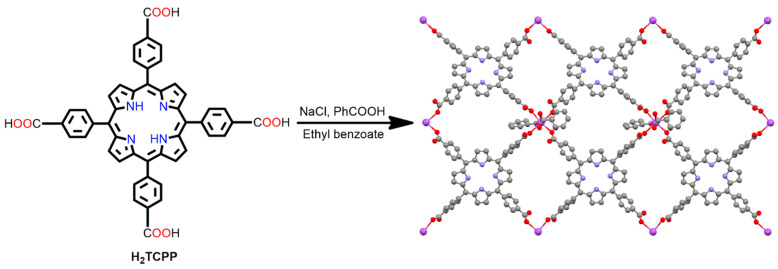
“Wavy” H_2_TCPP open frameworks within [Na^+^·(H_2_TCPP)·anion^−^]_n_. Adapted from Ref. [90].

Free H_2_TCPP or metalated MTCPP ligands have been used as ingredients for the fabrication of several P-MOFs. In 2000, Goldberg et al. reported an interesting Na-MOF composed of H_2_TCPP and Na^+^ ions [90]. Typically, the reaction of H_2_TCPP with NaCl in a mixture of benzoic acid, ethyl benzoate, and methanol leads to the formation of a 2D MOF associated with Na^+^ ions. Na-MOF consists of square-planar Na^+^(–COOH)_4_ synthons, in which each Na^+^ is coordinated to the COOH group of adjacent H_2_TCPP ligands. Benzoic acid plays a vital role in the self-assembly of H_2_TCPP as the benzoate ion imposes on the layered porphyrin topology by approaching the Na^+^ ion from the opposite side of the square-planar Na^+^(–COOH)_4_ moiety (Figure 11).

**Figure 12:**
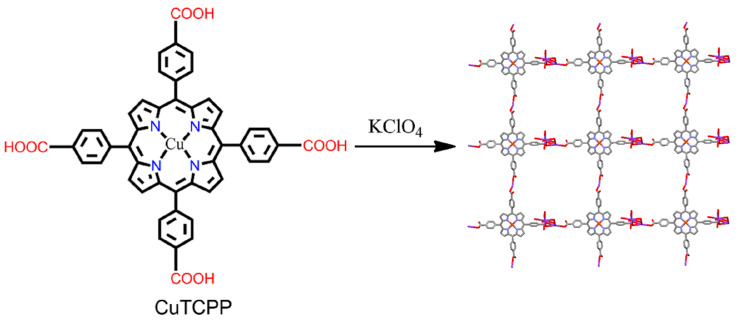
Offset-stacked frameworks in [K^+^·(CuTCPP)·solvated]_n_. Reproduced from Ref. [91].

The same group reported robust 3D MOFs based on CuTCPP ligands and K^+^ [91]. These compounds were synthesized by reacting CuTCPP with KClO_4_ in a mixture of nitrobenzene and methanol. The 3D MOF self-assembled by the association of the anionic porphyrin unit of CuTCPP with a binuclear {K^+^_2_(-COO^−^)_2_-(-COOH)_6_} moiety (Figure 12). A four-coordinate copper atom with no axial coordination in CuTCPP was offset-stacked with an inter-porphyrin distance of 3.87 Å.

**Figure 13:**
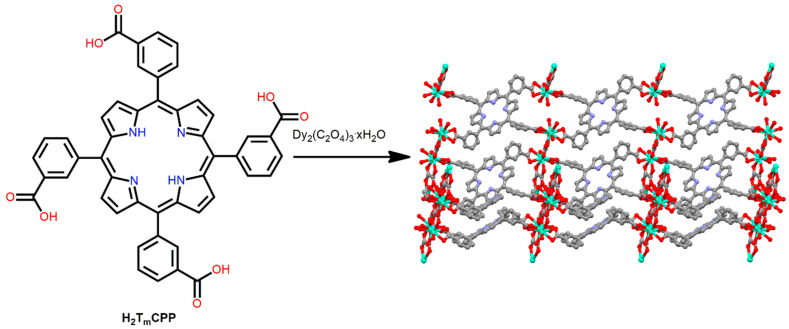
Partial view of the topology of Dy-MOF constructed from Dy_2_(C_2_O_4_)_3_·xH_2_O and H_2_T_m_CPP. Redesigned from Ref. [92].

A P-MOF was assembled by reacting H_2_TmCPP (5,10,15,20-tetrakis(m-carboxyphenyl)porphyrin) with Dy^3+^ ions [92]. Dy-MOF was fabricated from the reaction of H_2_TmCPP, Dy_2_(C_2_O_4_)_3_·xH_2_O, and a catalytic amount of pyridine in DMF under refluxing conditions (Figure 13).

A base was used to enhance the deprotonation of the carboxylic acid group in H_2_TmCPP. The Dy^III^ ion is a “hard” center and favors the construction of polynuclear building blocks linked by oxalate anions to form a “chair-like” conformation. The H_2_T_m_CPP in Dy-MOF exhibits a chair-like conformation, with two adjoining carboxylate groups directed downward and the other two upward. This study demonstrated a platform for the fabrication of a more porous and uniform framework utilizing lanthanide-bridging reagents with various porphyrin carboxylic acid ligands.

**Figure 14:**
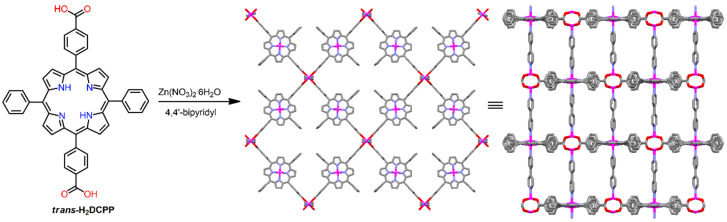
Structure of 2D porphyrinic grid fabricated from Zn(NO_3_)_2_·6H_2_O, H_2_DCPP, and 4,4′-bipyridyl. Adapted from Ref. [93].

In 2009, Choe et al. demonstrated a stable P-MOF from the reaction of Zn(NO_3_)_2_·6H_2_O, H_2_DCPP ([5,15-di(4-carboxyphenyl)-10,20-diphenylporphyrin])*,* and 4,4′-bipyridyl in DMF under solvothermal conditions [93]. Octahedral Zn porphyrinic paddlewheel clusters were assembled using bpy linkers to form a rare T-shaped anatase (ant) topology (Figure 14). Therefore, porphyrin connectors can be used to fabricate interesting node geometries comprising various organic linkers.

**Figure 15:**
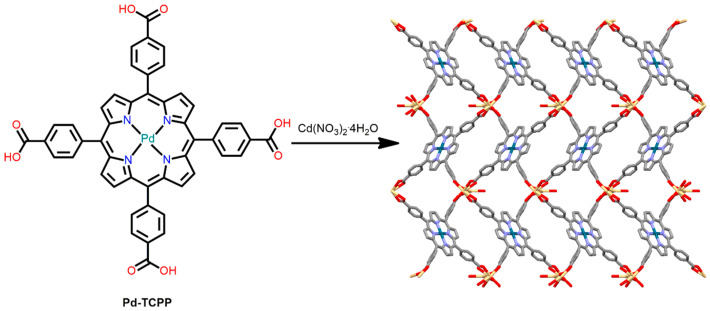
Underlying network topology of porphyrin-based MOF derived from Pd-TCPP and Cd(NO_3_)_2_·4H_2_O. Solvent molecules are excluded for clarity. Adapted from Ref. [94].

Wu et al. reported a 3D-MOF derived from the reaction of Cd(NO_3_)_2_·4H_2_O and Pd-TCPP in DMF under solvothermal conditions [94]. The crystal structures confirmed the presence of two crystallographically independent Cd^II^ centers in the unit cell. One Cd^II^ ion is linked to the eight carboxylate oxygen atoms of four adjacent Pd-TCPP molecules. In contrast, the second Cd^II^ ion is encircled by four carboxyl groups from four adjacent Pd-TCPP and two H_2_O molecules. Moreover, each Pd-TCPP molecule behaves as an octadentate ligand to coordinate with eight Cd atoms from four adjacent Cd clusters to form a 3D framework (Figure 15). This robust porous framework exhibits the selective oxidation of styrene in high yield. Pd^2+^ provides the active sites dispersed in the pores of the MOF, and the conjugated π-electron porphyrinic moiety facilitates the transfer of electrons during the reaction.

**Figure 16:**
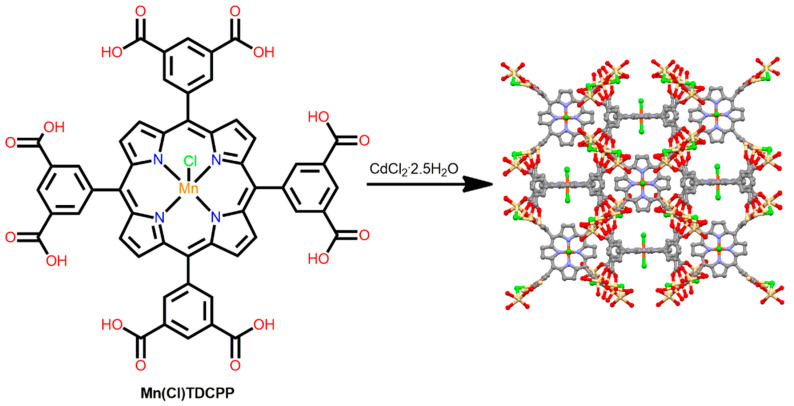
Topology of the MOF constructed from Mn(Cl)TDCPP and CdCl_2_·2.5 H_2_O. Adapted from Ref. [95].

A porous metalloporphyrinic framework derived from a tetra-substituted octatopic porphyrin 5,10,15,20-tetrakis(3,5-biscarboxylphenyl)porphyrin (H_2_TDCPP) linker was constructed from the reaction of Mn(Cl)TDCPP, CdCl_2_·2.5 H_2_O, and acetic acid in DMF [95]. The octa-carboxylate metallo-ligand Mn(Cl)TDCPP is linked by two secondary building units (a trinuclear cadmium cluster of carboxylate ligands, Cd_3_(COO)_4_Cl_2_(H_2_O)_4_, and a binuclear cadmium cluster of carboxylate ligands, Cd_2_(COO)_4_(H_2_O)_2_), forming 3D porous networks with tbo topologies (Figure 16). Several substrates were easily incorporated into the cavity of this material, confirming the porous framework of the MOF. This MOF exhibits outstanding photocatalytic performance and selectivity for the oxidation of alkylbenzenes. Mn^3+^ provides a reactive site, and the porphyrin core facilitates electron transfer during the reaction. Moreover, the specific pore size of this MOF provides selectivity for organic transformations.

**Figure 17:**
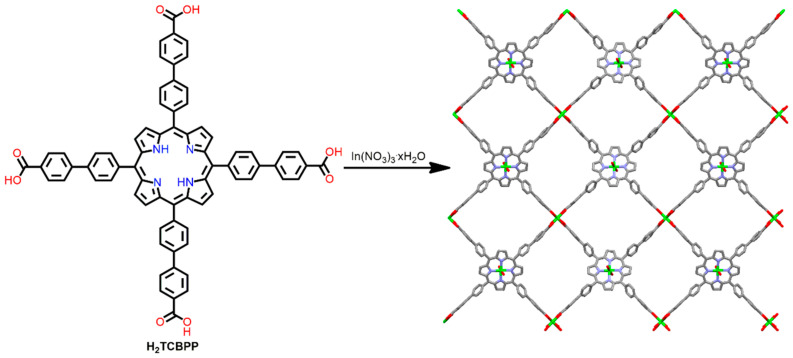
Structure of In^III^-MOF derived from H_2_TCBPP ligand and In(NO_3_)_2_·xH_2_O. Adapted from Ref. [96].

Ma et al. demonstrated a robust In^III^-MOF based on a custom-designed porphyrin tetra-carboxylate ligand [96]. The one-step solvothermal reaction of In(NO_3_)_3_·xH_2_O with H_2_TCBPP (5,10,15,20-tetrakis(4-carboxybiphenyl)porphyrin) in DMF leads to the generation of a 3D MOF with pts topology (4,4-linked binodal nets). In the case of In^III^-MOF, the asymmetric unit comprises four TCBPP^6−^ linkers and eight In^3+^ ions. Of the eight In^3+^ ions, four are situated in the porphyrin, and the other four are eight-coordinated. This In-MOF exhibited an N_2_ uptake capacity of more than 150 cm^3^ g^−1^ at 1 atm (Figure 17). For comparison, In^III^-MOF derived from H_2_TCPP ligands was prepared. Because of the smaller pore size in this case, no significant uptake of N_2_ was observed at 77 K. However, the former material shows a higher CO_2_ uptake capacity at 273 K (82 cm^3^ g^−1^) compared to the latter (55 cm^3^ g^−1^).

**Figure 18:**
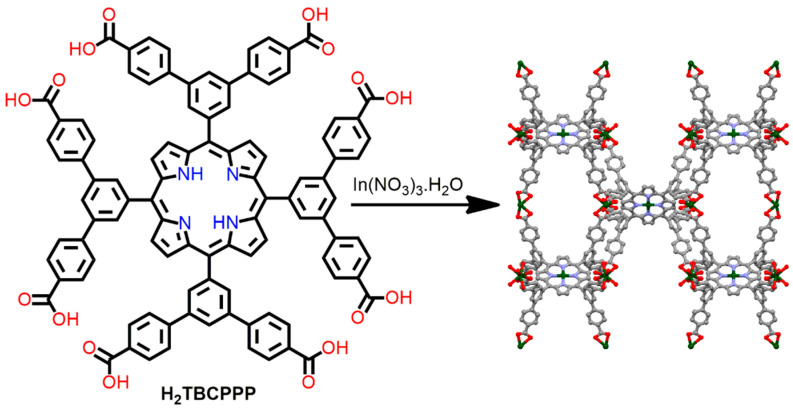
Topology of the MOF constructed from In(NO_3_)_3_·H_2_O and H_2_TBCPPP. Adapted from Ref. [97].

An anionic indium porphyrin framework composed of rare Williams β-tetrakaidecahedral cages was reported. The reaction of 5,10,15,20-tetrakis(3,5-bis[(4-carboxy)phenyl]phenylporphyrin (H_2_TBCPPP) and In(NO_3_)_3_·H_2_O in DMF under refluxing conditions leads to the formation of an In-MOF [97]. In this In-MOF, the octatopic ligand H_2_TBCPPP is coordinated with four-connected secondary building units [In(COO)_4_]^−^ (Figure 18). This In-MOF exhibited effective photocatalytic activity for the oxidation of aryl sulfides in the presence of air under LED irradiation. Interestingly, the catalytic activity could be controlled by altering the metal-ion content of the MOF. The high degree of metallization of In-MOF produces more reactive oxygen species and enhances catalytic performance. Therefore, this study demonstrated that the photoredox catalytic activity of porphyrin-based MOFs can be controlled. 

**Figure 19:**
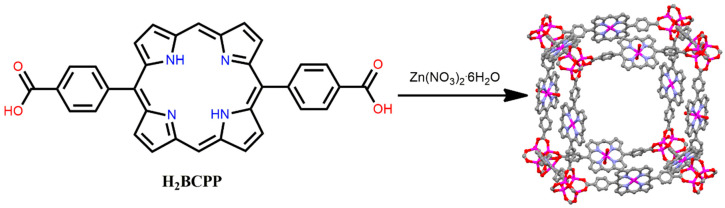
Structure of P-MOF derived from H_2_BCPP and Zn(NO_3_)_2_·6H_2_O. Adapted from Ref. [98].

Ditopic dicarboxylate porphyrin ligands, such as 5,15-bis(4-carboxyphenyl)porphyrin (H_2_BCPP), can be used for the fabrication of P-MOFs, such as tetratopic H_2_TCPP ligands. A primitive cubic (pcu) topology-based P-MOF was constructed from the reaction of a linear organic porphyrin H_2_BCPP and Zn(NO_3_)_2_·6H_2_O in DMF under solvothermal conditions [98]. Coordination between the tetranuclear zinc cluster Zn_4_(μ_4_-O)(−COO)_6_ and H_2_BCPP leads to the formation of four-fold interpenetrated P-MOF. Additionally, strong π-π stacking from H_2_BCPP ligand with Zn-carboxylate clusters generates a four-fold interpenetrating mesoporous framework. A BET surface area of 700 m^2^ g^−1^ at 77 K confirms the porous channel present within the porphyrin network (Figure 19). The micropores in this framework selectively exhibited moderate CO_2_ uptake over CH_4_. This behavior resulted in the size-selective chemical transformation of CO_2_ into cyclic carbonates in the presence of epoxides.

**Figure 20:**
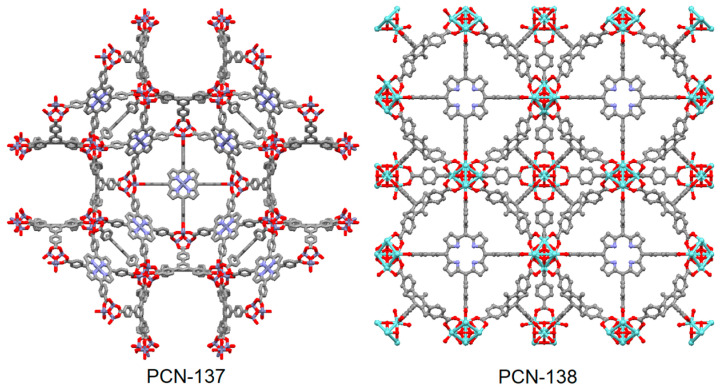
Structures of PCN-137 (7-connected Zn_4_O cluster) and PCN-138 (12-connected Zr_6_ cluster). Adapted from Ref. [99].

Two mixed-ligand face-sharing Archimedean polyhedron-based MOFs were constructed [99]. Refluxing the mixtures of Zn(NO_3_)_2_·6H_2_O, 1,3,5-Tris(4-carboxyphenyl)benzene (H_3_BTB), and H_2_TCPP in DMF leads to the formation of PCN-137. Additionally, PCN-138 was fabricated via the reaction of ZrCl4, 4,4′,4″-(2,4,6-trimethylbenzene-1,3,5-triyl)tribenzoic acid (H_3_TBTB), benzoic acid, and H_2_TCPP under reflux conditions. The metal clusters serve as vertices (ZnTCPP in PCN-137 and H_2_TCPP in PCN-138), and the tritopic carboxylate linkers act as the faces of the polyhedra (H_3_BTB in PCN-137 and H_3_TBTB in PCN-138) in these two 3D MOFs (Figure 20). PCN-138 showed better catalytic CO_2_ reduction activity under visible-light irradiation than PCN-137. This report suggests that the combination of polycarboxylate porphyrin ligands with various linkers of appropriate sizes can create complicated networks with interesting geometries.

### 2.3. Other Miscellaneous Porphyrinic MOFs

**Figure 21:**
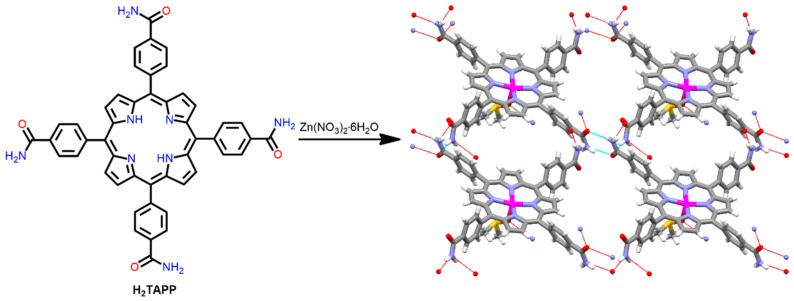
Structure of 2D ZnP-based supramolecular polymer constructed from the reaction of H_2_TAPP and Zn^2+^ ion. Adapted from Ref. [100].

In 1998, Goldberg and co-workers demonstrated a 2D porous network arising from the reaction of Zn(NO_3_)_2_·6H_2_O and 5,10,15,20-tetrakis(4-amidophenyl)porphyrin (H_2_TAPP) in dimethyl sulfoxide under refluxing conditions [100]. Hydrogen bonding between the adjacent CONH_2_ of H_2_TAPP leads to the formation of two-dimensional arrays parallel to the porphyrin plane (Figure 21). The square tetracarboxamide groups and their hydrogen bonding ability can be used to fabricate rigid and planar frameworks.

**Figure 22:**
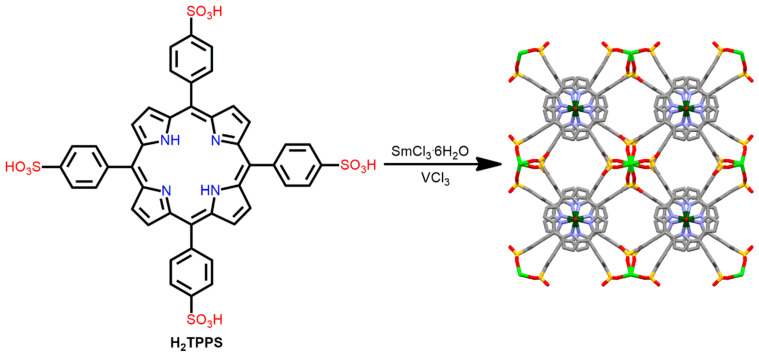
Structure of P-MOF derived from H_2_TPPS ligand, VCl_3_, and SmCl_3_·6H_2_O under solvothermal conditions. Adapted from Ref. [101].

Fukuzumi et al. reported a heterometallic P-MOF derived from 5,10,15,20-tetrakis(4-sulfonatophenyl)porphyrin (H_2_TPPS), which is a sulfonyl-based porphyrin linker [101]. The heterometallic P-MOF was constructed from the reaction of H_2_TPPS with VCl_3_ and SmCl_3_·6H_2_O under hydrothermal conditions. The tetratopic vanadium porphyrin linked to the secondary building blocks of [Sm(-SOO_2_)_4_] leads to the formation of a face-sharing MOF. The Sm ion is coordinated to eight O atoms from eight different sulfonate groups. Moreover, weak V═O···V interactions play a vital role in the 1D channel of porphyrins (Figure 22). The porous nature of this MOF was characterized by significant N_2_ gas adsorption at room temperature under cryogenic conditions. The relevant N_2_-encapsulated MOF structure was also established by crystallographic measurement.

**Figure 23:**
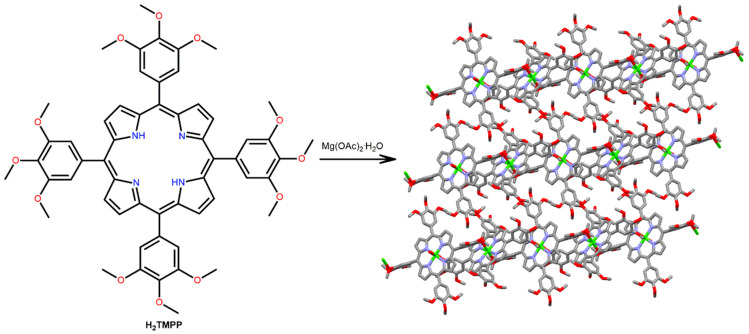
Structure of the 1D Mg-porphyrin-based coordination polymer derived from the reaction of Mg(NO_3_)_2_·H_2_O with H_2_TMPP. Adapted from Ref. [102].

Sarkar et al. reported a one-dimensional coordination framework obtained from the solvothermal reaction of Mg(NO_3_)_2_·H_2_O with 5,10,15,20-tetrakis(,4,5-trimethoxyphenyl)porphyrin (H_2_TMPP) in DMF [102]. The Mg-MOF exhibits unusual 1D coordination polymer networks arising from an axial Mg–O bond (O atom from the *m*-methoxy group of the neighboring H_2_TMPP molecule) (Figure 23). The Mg-MOF readily self-assembled in DCM/petroleum ether into spherical shapes and rectangles in ethanol.

**Figure 24:**
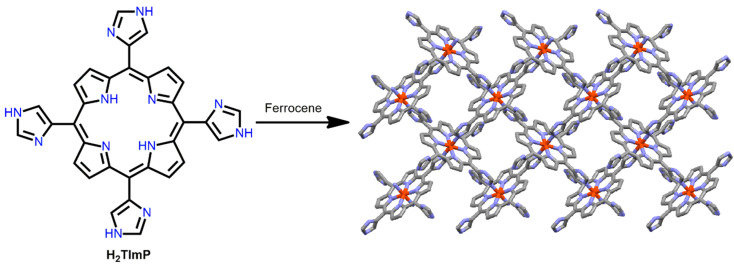
Structure of the 2D lamellar network of Fe-MOF. Redesigned from Ref. [103].

Liu et al. demonstrated a highly stable 2D Fe-MOF [103]. The solvothermal reaction of ferrocene with *meso*-tetra(4-imidazoyl)porphyrin (H_2_TImP) in DMF leads to the formation of a robust 2D Fe-MOF (Figure 24). This 2D lamellar framework is stable in air, common solvents, and saturated NaOH solution. The strong proton affinity of the imidazole ligand is responsible for its enormous stability under alkaline conditions. Another reason is that the strong Fe^3+^–imidazolate bond prevents further dissociation from the robust frameworks. Therefore, this report suggests that an ultra-strong metal coordination framework is possible through the reaction of high-valence metal ions and imidazole-based ligands.

**Figure 25:**
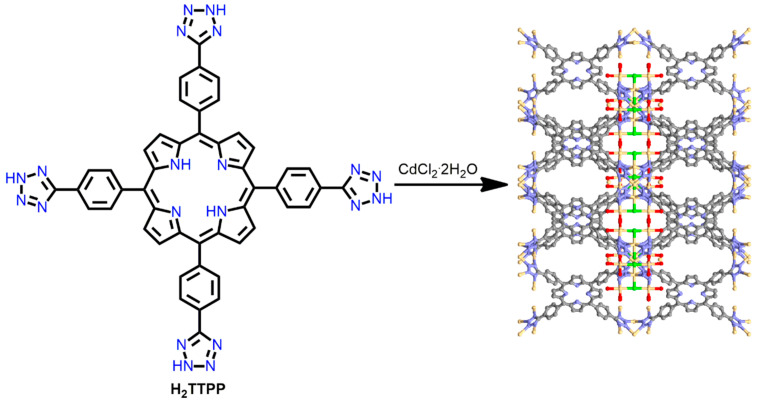
Intersection frameworks in Cd-MOF. Adapted from Ref. [104].

In addition to pyridyl-metal linkages, metal-azolate coordination bonds have also been explored for the fabrication of P-MOFs. For example, robust Cd-MOF was synthesized from the reaction of CdCl_2_·2H_2_O with 5,10,15,20-tetrakis [4-(2H-tetrazol-5-yl)phenyl]porphyrin (H_2_TTPP) in methanol and DMA (*N*,*N*′-dimethylacetamide) under refluxing conditions [104]. In Cd-MOF, eight μ_2_-tetrazole ligands in H_2_TTPP are connected with square planar chloride-centered [Cd_4_Cl]^7+^ moieties to form a 3D network with scu topology (Figure 25). The Cd-MOF showed single-crystal-to-single-crystal conversion upon changing the temperature. The 1D channel underwent modification from a square to a rectangular structure when it cooled from 298 K to 110 K, and vice versa. This distinctive phase transition property has been exploited in the construction of sensing devices.

**Figure 26:**
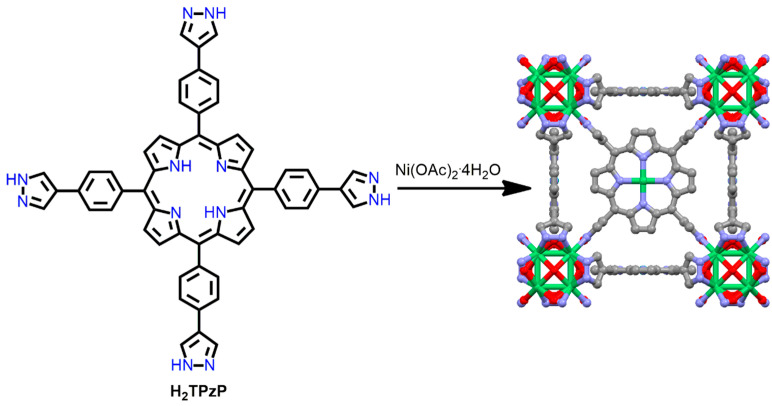
Geometrical view of 3D frameworks in Ni-MOF. Adapted from Ref. [105].

Zhou et al. fabricated a Ni-MOF based on pyrazole-metal coordination [105]. Ni-MOF was integrated from the refluxing of the mixture of Ni(OAc)_2_·4H_2_O and 5,10,15,20-tetra(1*H*-pyrazol-4-yl)porphyrin (H_2_TPzP) in the presence of triethylamine in DMF (Figure 26). The four pyrazole ligands were connected to the [Ni_8_] clusters to form 3D porous networks. Furthermore, the porosity and framework structure of the Ni-MOF were not influenced by submergence in boiling, saturated NaOH solutions. The Ni-MOF framework exhibited an outstanding porosity of 1309 m^2^/g in an extremely basic medium. Owing to the strong affinity between protons and pyrazolate groups, Ni^+2^ ions afford relatively stable frameworks with highly basic N-donor pyrazolate linkers. Therefore, the Ni-MOF exhibits excellent reusability and can be used for photocatalytic water remediation in strongly basic media.

## 3. P-MOF Materials for Photocatalytic Treatment of Wastewater

In general, photocatalysts are excited by the absorption of a photon (*hv*) with energy greater than E_g_ (band gap energy) [106,107]. This operation generates a charge separation due to the transfer of an electron from the VB (valence band) to the CB (conduction band), thus generating a pair of reactive species (h^+^ in the VB and e^−^ in the CB). A photocatalytic reaction proceeds if the recombination of hole pairs is delayed. This is because excited electrons react with the dye molecule to create a reduced product, and excited holes react with the dye molecule to generate an oxidized product. Alternatively, the activated electrons can react with O_2_ (the electron acceptor) dissolved in an aqueous solution and reduce it to a superoxide radical anion (O_2_^•−^). On the other hand, the excited holes can react with OH^−^ or water and oxidize them into hydroxyl radicals (^•^OH). Other highly oxidizing materials, such as peroxide radicals, may also be produced during photodecomposition. The O_2_^•−^ is oxidized by the hole in the photocatalyst and partially becomes a singlet oxygen molecule (^1^O_2_). The resulting ROS are strong oxidizing agents that can mineralize pollutants into less toxic molecules. Based on these assumptions, the relevant reactions on the surface of the photocatalyst can be summarized as follows:

P + *hν* → P^*^(h^+^_VB_ + e^−^_CB_) (1)

P^*^ (h^+^_VB_) + H_2_O → H^+^ + ^•^OH + P (2)

P^*^ (h^+^_VB_) + OH^−^ → ^•^OH + P (3)

P^*^ (e^−^_CB_) + O_2_ → O_2_^•−^ + P (4)

H^+^ + O_2_^•−^ → HO_2_^•^ (5)

P^*^ (h^+^_VB_) + O_2_^•−^ → ^1^O_2_ (6)

Dye + h^+^_VB_ → oxidation products (7)

Dye + e^−^_CB_ → reduction products (8)

Dye + ^•^OH → degradation products (9)

Dye + O_2_^•−^ → degradation products (10)

Dye + ^1^O_2_ → degradation products (11)

where *hν* is the energy required to promote electrons from the VB to the CB.

Moreover, the catalytic activity of photocatalysts depends on several important factors, including the E_g_, electron–hole recombination rate, surface morphology, crystallinity, phase composition, light-harvesting ability, permanent porosity, and adsorption capacity of the contaminants on the surface of the photocatalysts. Owing to their extensive morphology, photofunctional properties, and high photosensitivity, P-MOFs have been widely employed for the degradation of pollutants in wastewater [72,73,74]. P-MOFs can be readily fabricated, and their structural framework can be easily controlled. Therefore, tuning the conformational structure of P-MOFs positively affects their permanent porosity, light-harvesting ability, and catalytic active sites. Additionally, intense electronic delocalization of the photogenerated reactive species on the surface of π-conjugated porphyrin molecules delays the recombination process and hence improves their photodegradation performance. Their photodegradation capacity is controlled not only by their structural topology but also by their surface morphology. Because of intense π-π interactions in *J* or *H*-type interactions, P-MOFs act as superb semiconductor materials and can enhance charge separation during the photodegradation reaction. In general, P-MOF materials absorb photon energy from solar light, and electrons in the VB are transferred to the CB, creating hole–electron pairs. These hole–electron pairs participate in the photodegradation process to produce reactive oxygen species and oxidize toxic contaminants to non-toxic CO_2_ and H_2_O. Excellent electronic delocalization of these photogenerated charge species occurs on the surfaces of the P-MOF materials. This delays the recombination of photogenerated species (e^−^ and h^+^) and thus enhances the photodegradation activity. The permanent porosity inside the cavities of P-MOFs provides the space required for guest molecule encapsulation. In addition, the large micropores provide a large number of active sites (arising from metal ions) for interactions with many types of cationic, anionic, and neutral dyes. These robust P-MOFs not only harvest photons but also prevent the decomposition of reactive species during the degradation process and increase recyclability. Each P-MOF material differs from the other in terms of morphology, light-harvesting ability, chemical stability, and degradation ability. Therefore, the catalytic photodegradation properties are controlled by the structural geometry of the P-MOF materials.

Radical trapping experiments (scavenger tests or electron spin resonance spectroscopy) are used to detect the ROS generated during photocatalysis. Generally, *tert*-butanol (^t^BuOH) has been used to seize ^•^OH, Na_2_-EDTA (ethylenediaminetetraacetic acid disodium) has been used to capture h^+^, *p*-BQ (*para*-benzoquinone) has been used for O_2_^•−^, and NaN_3_ (sodium azide) has been utilized for ^1^O_2_ during the photodegradation process [108,109].

**Figure 27:**
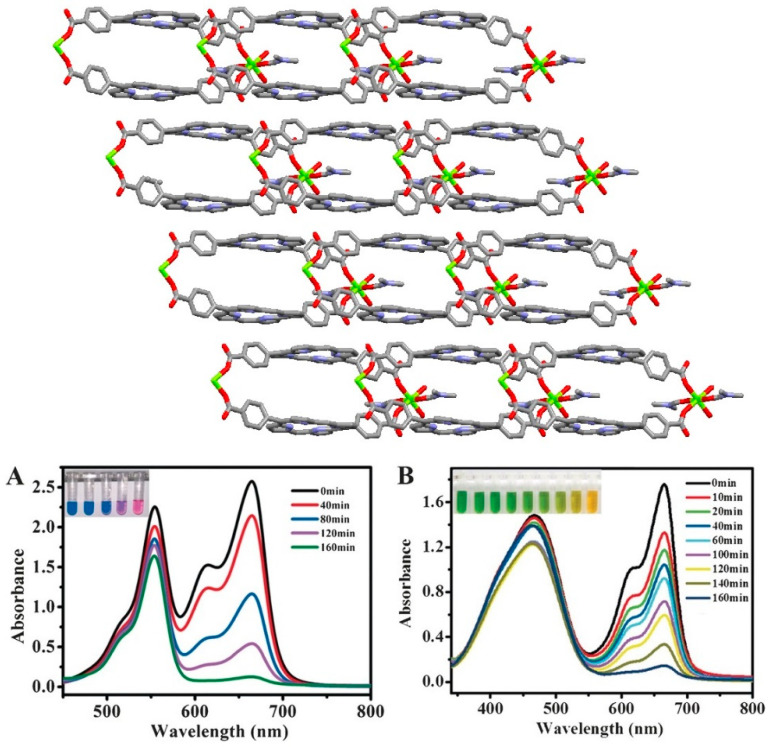
Topology of mutually staggered conformation of H_2_DCPP ligand in Ba-MOF frameworks. UV/Vis spectral change in RhB and MB dyes (**A**); and MO and MB dyes (**B**) in the presence of Ba-MOF. Adapted from Ref. [110].

Jiang and co-workers demonstrated a highly porous porphyrin-based 3D Ba-MOF derived from the reaction of BaCl_2_·6H_2_O with H_2_DCPP in DMA under solvothermal conditions [110]. Each Ba^II^ ion is surrounded by eight oxygen atoms. Of the eight oxygen atoms, six come from the six carboxyl oxygen atoms of the six adjacent H_2_DCPP linkers, and the remaining two come from two solvent molecules (H_2_O and DMA). The two adjacent Ba^II^ ions are connected to a Ba^II^ cluster by the carboxyl oxygen atom of the H_2_DCPP ligands and are also attached to a 3D porous network via the terminal carboxyl groups of the adjacent H_2_DCPP linker (Figure 27). This Ba-MOF shows good consumption capacity of MB (methylene blue) dye (~306 mg g^−1^). The electrostatic interactions between the cationic MB dye and the uncoordinated pyrrole N atoms of the porphyrin molecules facilitate high dye consumption. Furthermore, this material exhibits outstanding selectivity for the removal of MB over RhB (rhodamine B) or MO (methyl orange) (Figure 27). The pore sizes of the Ba-MOF framework facilitate the encapsulation of smaller MB dyes over larger dyes. This report predicts that porous P-MOFs have the potential to destroy toxic dyes in wastewater.

**Figure 28:**
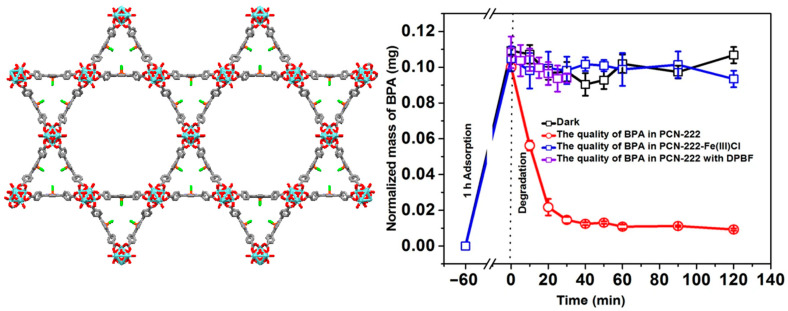
Topology of PCN-222(Fe) frameworks. Decomposition curve of bisphenol A in the presence of PCN-222(Fe). Reproduced from Ref. [111].

Robust PCN-222(Fe) has been utilized for the degradation of bisphenol A (BPA) [111]. PCN-222(Fe) comprises the Zr6 cluster and the FeTCPPCl ligand (Figure 28). It can be obtained by reacting benzoic acid, ZrCl_4_, and FeTCPPCl in DMF under refluxing conditions [112] and has outstanding stability in water and concentrated acids.

Mesoporous PCN-222(Fe) exhibits excellent catalytic activity because the active sites are positioned inside a cavity with an open channel. Thus, PCN-222(Fe) can be used to remove pollutants from wastewater. It shows excellent capacity to absorb BPA (487.69 ± 8.37 mg g^−1^) and an ultrahigh ability to degrade BPA from aqueous solutions (photodegradation rate = 0.004 mg min^−1^ within 20 min). ^1^O_2_ was generated during the visible-light irradiation of PCN-222(Fe) and acted as a reactive oxygen species to degrade BPA molecules. Therefore, the permanent porosity and interaction of BPA molecules with the active sites of PCN-222(Fe) can accelerate the catalytic process (Figure 28). However, after 2 h of visible-light irradiation, the rate of degradation was found to decrease because of the formation of *p*-benzoquinone (a single oxygen scavenger). Moreover, the addition of oxidants increased the photocatalytic efficiency of the reaction.

**Figure 29:**
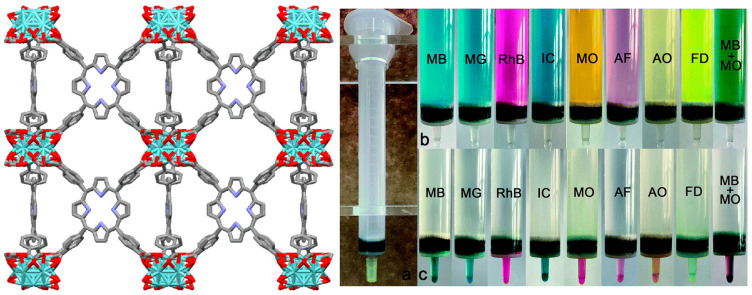
Structure of PCN-222. (**a**) A syringe filled with PCN-222. (**b**) Various dyes or their mixtures loaded into syringes. (**c**) Dyes cleansed off by saturated NaCl/DMF or 0.1 M HCl/methanol. Adapted from Ref. [113].

PCN-222, a porphyrin-containing Zr-MOF, exhibits outstanding adsorption and removal efficiencies for several cationic and anionic dyes [113]. PCN-222 is fabricated from the reaction of ZrOCl_2_·8H_2_O with H_2_TCPP in DMF under solvothermal conditions. In PCN-222, each cube consists of eight corner-sharing Zr_6_O_4_(OH)_4_ clusters and six face-sharing H_2_TCPP organic linkers. Additionally, each H_2_TCPP ligand bridges four Zr_6_O_4_(OH)_4_ blocks. The BET surface area of PCN-222 was found to be 2336 m^2^ g^−1^, with a large pore size of 3.2 nm. This confirms that PCN-222 possesses permanent porosity, which enables the quick adsorption and easy desorption of dyes. In addition, PCN-222 shows a zeta potential (ζ) of 23.5 to −13.6 mV in the pH range of 3 to 10, with an isoelectric point at pH 8. Under an acidic pH, PCN-222 showed a positive zeta potential and adsorbs anionic dyes. Electrostatic interactions between protonated MOFs and anionic dyes are responsible for high absorption. On the other hand, under a basic pH, PCN-222 showed a negative zeta potential and adsorbs the cationic dyes. This is due to the π-π interaction and electrostatic interaction between deprotonated MOFs and cationic dyes. These characteristics of the push–pull mechanism indicate the dual function of PCN-222 for the adsorption of cationic dyes, anionic dyes, or even their mixtures (Figure 29).

**Figure 30:**
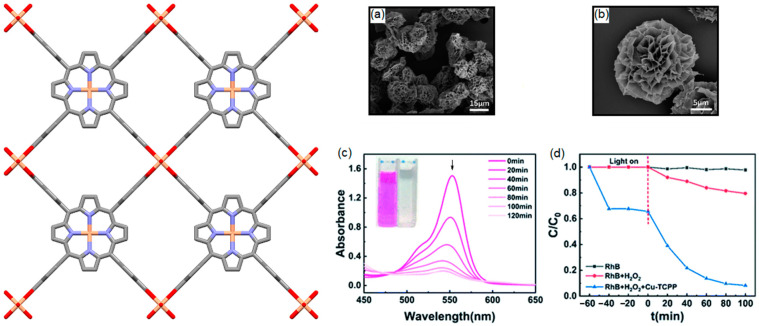
Images show a 2D topology of Cu-MOF derived from H_2_TCPP and Cu(NO_3_)_2_·3H_2_O. SEM images (**a**,**b**), UV-vis absorption spectrum of RhB dye (**c**), and decomposition curve (**d**) in the presence of Cu-MOF and H_2_O_2_. Adapted from Ref. [114].

Flower-shaped nanosheets of Cu-MOF, which has a 2D network, were fabricated from the reaction of Cu(NO_3_)_2_·3H_2_O and H_2_TCPP under solvothermal conditions [114]. In the presence of trifluoroacetic acid, these 2D nanosheets were converted into the flower-like nanostructure of Cu-TCPP. Trifluoroacetic acid was used to control the morphology of flower-like Cu-TCPP. This flower-like Cu-TCPP exhibited outstanding catalytic photodegradation of the RhB dye. This is because of its large surface area, large number of active sites, and robustness in water. This flower-like Cu-TCPP has high permanent porosity, as evident from the large BET of 605.04 m^2^ g^−1^. Its RhB dye removal efficiency was 88% within 100 min of visible-light irradiation (Figure 30).

**Figure 31:**
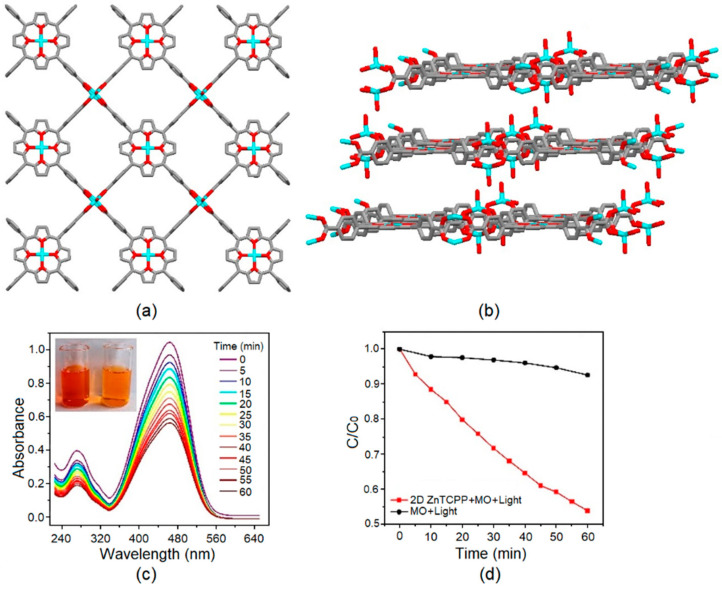
Structure of the 2D Zn-MOF along a-axis (**a**) and b-axis (**b**). Change in the UV-vis spectra of MO in the presence of Zn-MOF (**c**) and decomposition curve (**d**). Adapted from Ref. [115].

Tang and co-workers reported a highly porous 2D Zn-MOF derived from the solvothermal reaction of Zn(NO_3_)_2_·6H_2_O with H_2_TCPP in DMF [115]. This 2D-layered Zn-MOF was constructed by alternatively linking the four arms of the carboxylates of the ZnTCPP derivatives with Zn^2+^ (Figure 31).

The dislocated 2D ZnTCPP layers can successfully prevent self-quenching and generate large porous areas. The incorporation of Zn^II^ ions enhances spin-orbital coupling via the heavy-atom effect and facilitates the intersystem crossing process for the generation of singlet oxygen (^1^O_2_). Moreover, significant charge transfer between porphyrin ligands and Zn and the existence of active sites in ZnTCPP facilitate the production of a large amount of ROS, such as ·OH or ^1^O_2_, during the photodegradation process. These ROS can be utilized to quickly decompose the MO dye within 60 min. Furthermore, this Zn-MOF can degrade other pollutant dyes such as MB, RhB, or crystal violet (CV) via sonocatalysis (Figure 31).

**Figure 32:**
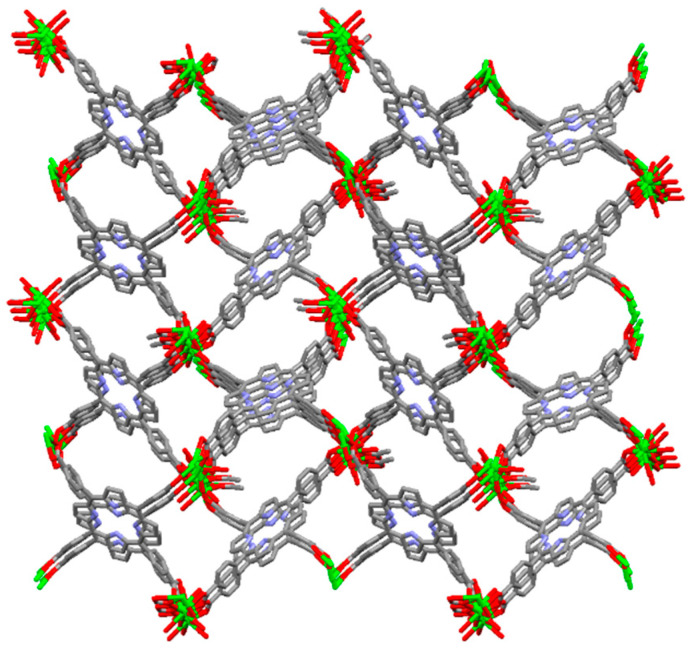
A 3D coordination framework without solvent molecules. Adapted from Ref. [116].

Chen et al. fabricated a porphyrin-containing Sr-MOF ([Me_2_NH_2_][Sr_2_(TCPP)(OAc)(H_2_O)]·2DMA) from the reaction of Sr(NO_3_)_2_, H_2_TCPP, and *N*-methyl-2-pyrrolidinone in DMA under refluxing conditions [116]. Two different coordination modes have been observed for Sr^2+^ in the Sr-MOF. In one case, Sr is connected to eight oxygen atoms in a twisted dodecahedral configuration, which originates from one acetate anion and seven carboxylate oxygen atoms of the H_2_TCPP linker. The second Sr atom is also coordinated with eight oxygen atoms from the SrO_8_ polyhedral cluster. The carboxylate group of H_2_TCPP provides five oxygen atoms, and the remaining three carboxylate oxygen atoms are from H_2_O and acetate (Figure 32).

**Figure 33:**
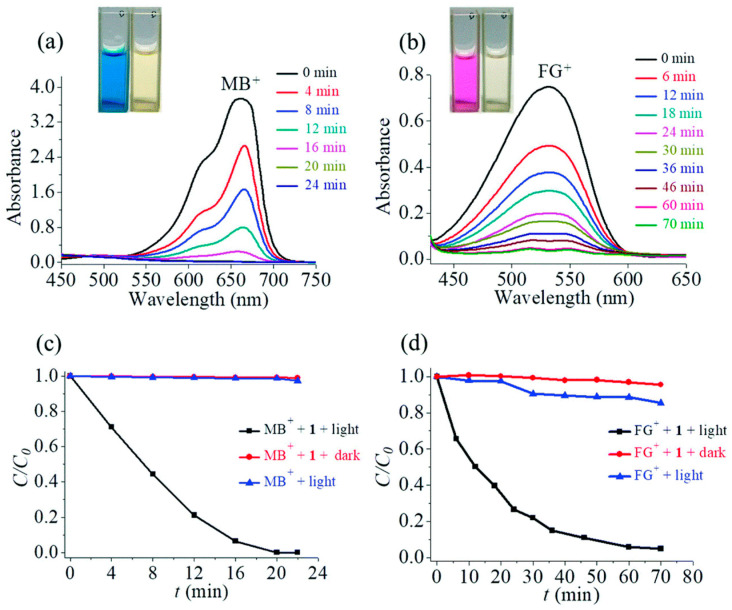
UV-vis absorption spectrum for the degradation of MB dye (**a**) and astrazone pink FG dye (**b**) with Sr-MOF. Degradation curve of MB (**c**) and FG (**d**). Redesigned from Ref. [116].

Interestingly, the cavity of this Sr-MOF has two types of 1D channels, one of which consists of Me_2_NH_2_^+^. This Sr-MOF showed excellent adsorption and photodegradation abilities against RhB and MB under visible-light irradiation. This is because Me_2_NH_2_^+^ can be reciprocated by cationic dyes (MB or FG) to enhance the adsorption of the pollutant dyes. The degradation activity exceeded 99% within 3 h for RhB and 99% within 22 min for MB (Figure 33). The main ROS for the cationic dye degradation process were found to be O_2_^•−^, ^1^O_2_, and h^+^.

**Figure 34:**
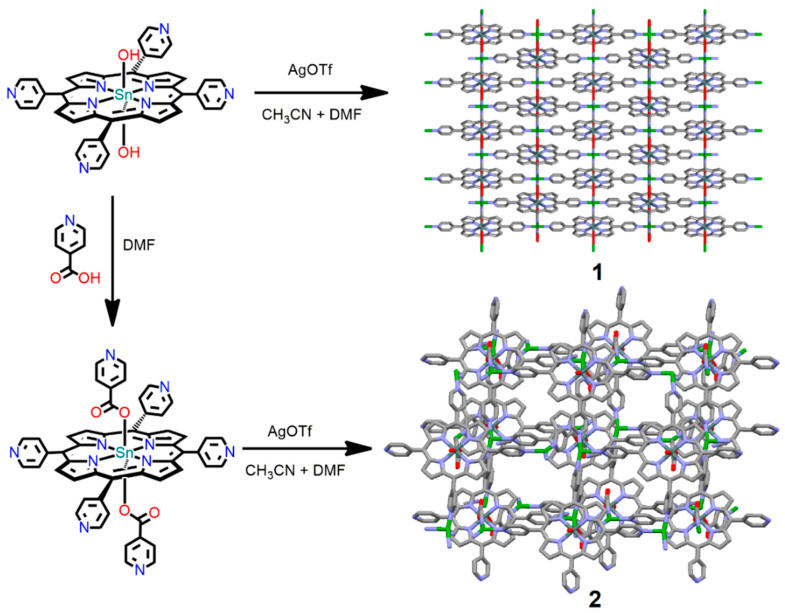
Structure of 2D coordination framework of **1** and 3D framework of **2** without the solvent molecules. Adapted from Ref. [117].

In 2022, Kim et al. reported two tin porphyrin-containing MOFs, [Ag_2_(TPyP)Sn(OH)_2_](NO_3_)_2_·(solv)_x_ (**1**) and [Ag_2_(TPyP)Sn(INA)_2_](OTf)_2_·(CH_3_CN)_2_ (**2**) (OTf = CF_3_SO_3_^−^; INA = isonicotinato anion), for the catalytic degradation of organic dyes [117]. These robust frameworks were prepared by the self-aggregation of six-coordinate tin porphyrin (*meso*-tetra-(4-pyridyl)porphyrinato)Sn(IV) coordinated with Ag(I) ions (Figure 34). The axial ligation of tin porphyrins not only controls the topology from 2D to 3D frameworks but also generates large conformational changes, including large permanent porosity, interesting morphology, robustness, and the catalytic degradation of pollutant dyes, such as MB, amaranth dye (AM), and bromocresol green (BCG).

By changing the axial substituent, the BET surface area increased 2.5-fold from **1** to **2**. The morphology also changed from fused particles (**1**) to flakes (**2**). This morphological change alters the surface energy and the number of active sites. Overall, this modification affects the photocatalytic activity. Because of the electronic interactions of pollutant molecules with the active sites of Ag-MOFs, the degradation rate constant for neutral dyes is lower than that for ionic dyes.

**Figure 35:**
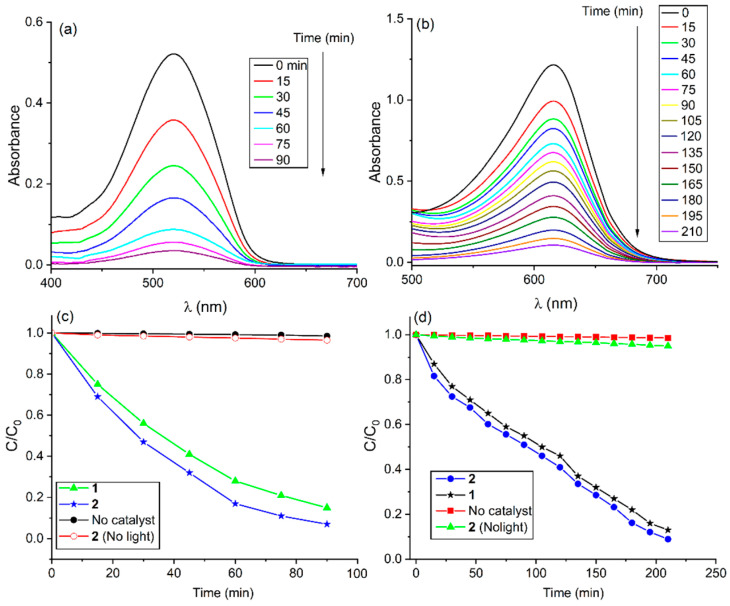
UV-vis absorption spectrum of AM dye (**a**) and BCG dye (**c**) in the presence of **1** and **2**. Decomposition curve of AM dye (**b**) and BCG dye (**d**). Adapted from Ref. [117].

The degradation rate constants for the dyes MB, AM, and BCG using catalyst **1** were found to be 0.015, 0.021, and 0.009 min^−1^, respectively, whereas the corresponding photodegradation rate constants using catalyst **2** were 0.020, 0.031, and 0.011 min^−1^, respectively. Thus, the catalytic degradation activity of **2** is better than that of **1** (Figure 35). Therefore, by tuning the axial substitution, MOFs with significantly improved catalytic efficiency against organic dyes can be fabricated.

**Figure 36:**
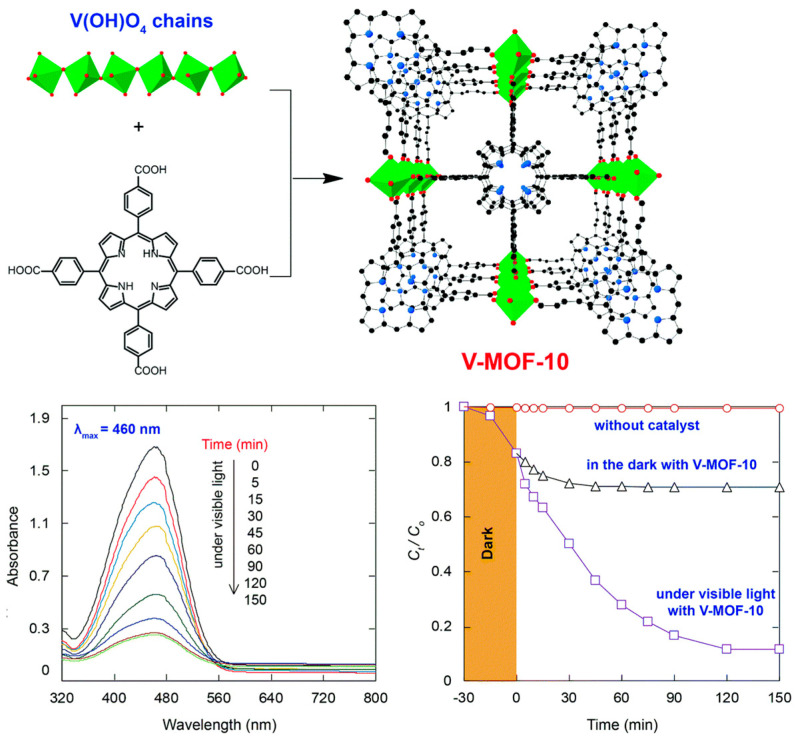
Structure of V-MOF-10. UV/Vis spectral change in MO dye and decomposition curve in the presence of V-MOF-10. Adapted from Ref. [118].

Nguyen et al. reported a P-MOF called V-MOF-10 [V_2_(OH)_2_(H_2_TCPP)] [118], which was prepared by reacting VCl_3_ with H_2_TCPP in DMF under solvothermal conditions (Figure 36).

Each carboxylate arm of H_2_TCPP is connected with {V(OH)O_4_}_∞_ secondary building units. V-MOF-10 shows high permanent porosity, as evident from the large surface area of 1477 m^2^ g^−1^ (BET). It also shows remarkable catalytic photodegradation of MO dye. Using UV-vis diffuse reflectance spectroscopy analysis, the optical band gap of V-MOF-10 was found to be 1.71 eV. Under visible-light irradiation, 89% of the MO dye could be removed within 120 min (Figure 36).

**Figure 37:**
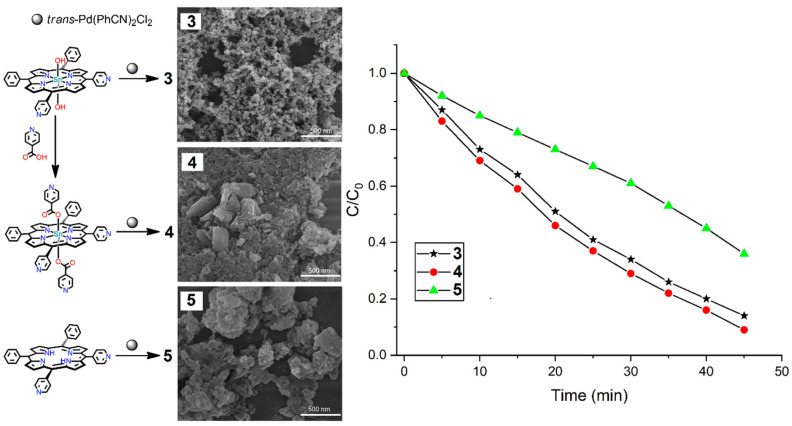
FE-SEM images of **3**, **4**, and **5**. Visible-light photodegradation of AO dye in the presence of **3**, **4**, and **5**. Adapted from Ref. [119].

Kim et al. recently reported a series of stable tin(IV)porphyrin-containing supramolecular frameworks (**3**–**5**) [119]. These were fabricated from the reaction of the *trans* isomer of Pd(PhCN)_2_Cl_2_ with three elementary porphyrin units (*trans*-dihydroxo)[5,10-bis(4-pyridyl)-15,20-bis(phenyl) porphyrinato]}tin(IV), *trans*-diisonicotinato)[5,10-bis(4-pyridyl)-15,20-bis(phenyl)porphyrinato]}tin(IV)), and 5,10-bis(phenyl)-15,20-bis(4-pyridyl)porphyrin, respectively. The conformational motifs in these networks change from 2D to 3D, influenced by the axial groups of the tin porphyrin moieties. The axial ligation of the Sn(IV)porphyrin moieties not only alters their conformational patterns (2D to 3D) but also remarkably changes their morphology, permanent porosity, and robustness. These conformational changes drastically affected the catalytic photodegradation of the AO (acid orange) dye under solar light irradiation. These photocatalysts removed 86–91% of the AO dye within 90 min in the presence of solar light. The degradation rate constants for catalysts **3**, **4**, and **5** were found to be 0.043, 0.047, and 0.021 min^−1^, respectively (Figure 37).

**Figure 38:**
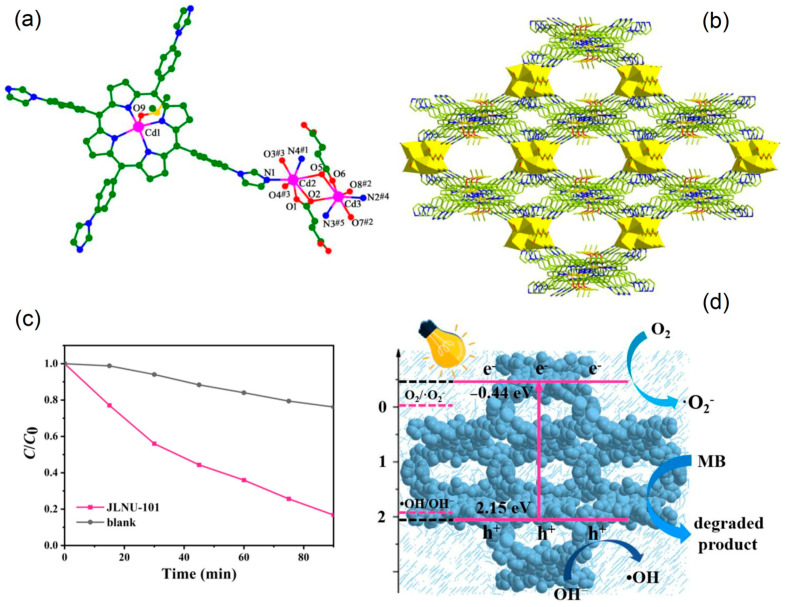
Coordination mode of Cd^2+^ in JLNU-101: (**a**) 3D framework. (**b**) Decay of MB dye in the presence of JLNU-1. (**c**) Possible degradation mechanism. (**d**) Adapted from Ref. [120].

Visible-light degradation of the MB dye was performed using a porphyrinic MOF (JLNU-101) [120]. The solvothermal reaction of Cd(NO_3_)_2_.4H_2_O and 5,10,15,20-tetrakis(4-(imidazol-1-yl)phenyl)porphyrin in the presence of fumaric acid leads to the formation of JLNU-101. In the crystal structure of JLNU-101, the [Cd_2_] nodes are coordinated by Cd^II^-porphyrin and external linker fumarate ligands, producing a 3D framework with an unfamiliar *fsc* topology. Owing to its solar-light-harvesting capacity and appropriate band energy level, JLNU-101 exhibited significant catalytic photodegradation performance against MB dye (83% of MB was degraded within 90 min) under LED light irradiation. Radical trapping experiments established that ^•^OH, O_2_^•−^, and h^+^ were the reactive species for the oxidation of MB dye (Figure 38).

**Figure 39:**
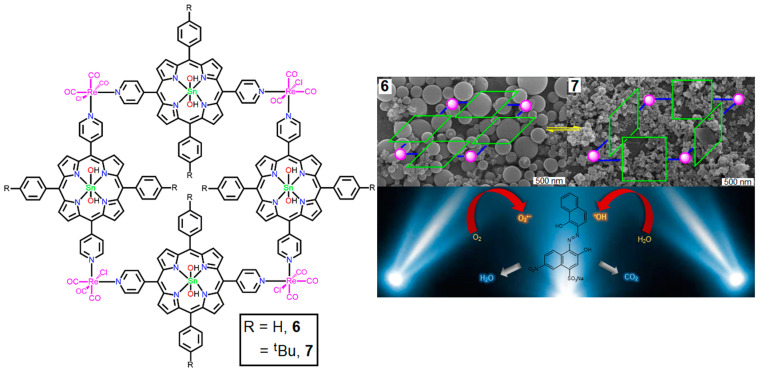
Tin porphyrin-based metallocycles **6** and **7** for photodegradation of EBT dye. Adapted from Ref. [121].

Re(I) metal-mediated P-MOFs **6** and **7** were used for the catalytic photodegradation of EBT (Eriochrome Black T) dye under visible-light irradiation [121]. Metallocycles **6** and **7** were fabricated by the reaction of Re(CO)_5_Cl with two Sn(IV)porphyrin-based building block units in mixed solvents (THF/Toluene = 1:1) under refluxing conditions. The conformational patterns of these frameworks were determined using closed 2D tetrameric arrays. Tuning the substituent on the building units not only changes the geometrical frameworks (horizontal to upright) but also creates high conformational changes, including high porosity, high thermodynamic stability, unique morphology, and excellent photodegradation capacity of the EBT dye (Figure 39).

These catalysts removed 88–95% of the EBT dye within 1.5 h of solar light irradiation. The photodegradation rate constant of **7** (0.032 min^−1^) is higher than that of **6** (0.023 min^−1^).

## 4. Hybrid Photocatalysts Based on P-MOFs for Water Remediation

Thus far, the discussion has been limited to the fabrication of porphyrin-based metal–organic frameworks from various porphyrinic compounds and the investigation of the photodegradation of toxic contaminants as free-standing organic semiconductors. However, during the photodegradation reaction, the photogenerated charge species (e^−^ and h^+^) either recombines or reaches the catalyst’s surface and facilitates a series of photodegradation steps. To improve the photodegradation performance in terms of solar energy conversion, permanent porosity, efficient charge separation, and recyclability, porphyrin-based metal–organic framework materials can be integrated with other photoactive semiconducting materials such as organic-based compounds (GO or g-C_3_N_4_) or inorganic-based semiconductors (TiO_2_, ZnO, or even metals). The incorporation of a second guest molecule into porphyrin-based framework materials has been found to not only enhance the photocatalytic activity but also improve the durability of P-MOFs under drastic reaction conditions.

**Figure 40:**
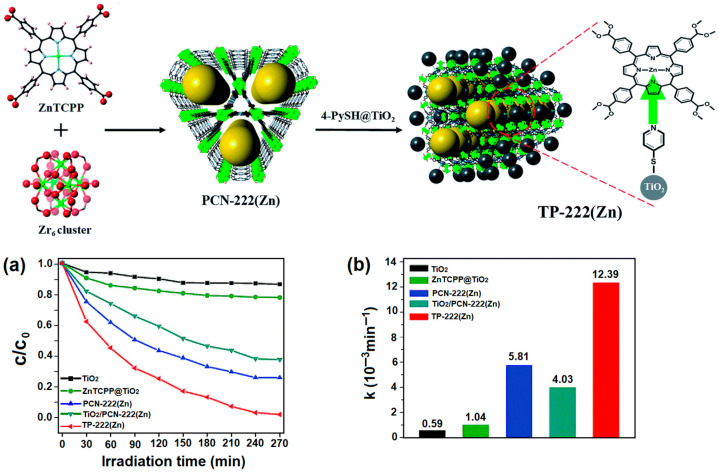
Synthesis of composite catalyst TP-222(Zn). (**a**) Photocatalytic removal of RhB dye with various photocatalysts. (**b**) Comparison of RhB degradation rate constants. Reproduced from Ref. [122].

Feng et al. reported the efficient fabrication of the hybrid catalyst TP-222(Zn) [122]. In the composite photocatalyst, TiO_2_ NPs are axially anchored to the Zn atom of PCN-222(Zn) via 4-PySH (4-mercaptopyridine). The incorporation of TiO_2_ nanoparticles successfully enhanced the lifetime of charge carrier recombination, facilitating electron transfer from PCN-222(Zn) to TiO_2_ nanoparticles, thus increasing the photodegradation efficiency of the RhB dye. In addition, the fabrication of this hybrid catalyst generates a large number of active sites, facilitating charge separation from the MOF to TiO_2_. The photocatalytic removal rate of RhB dye under visible-light irradiation was found to be 0.01239 min^−1^. Furthermore, the high porosity and interaction between the substrates within the active sites of the TP-222 (Zn) photocatalyst facilitate the degradation of organic contaminants. The composite TP-222(Zn) showed an efficient degradation capacity for RhB dye in the presence of solar light (Figure 40).

**Figure 41:**
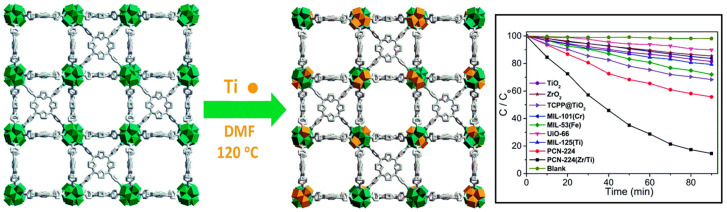
Structure of PCN-224(Zr/Ti). Decomposition curve of MB dye in the presence of various catalysts. Adapted from Ref. [123].

He et al. successfully added Ti as a metal node to Zr-MOF (PCN-222) using a simple and economical post-synthetic modification strategy [123]. In a typical process, PCN-222 is mixed with TiCp_2_Cl_2_ in DMF at 120 °C for cation exchange. After 48 h, the Zr-Ti exchange rate was 33.7%. UV-vis spectroscopy showed that the incorporation of Ti lowered the band gap from 1.86 eV for pure PCN-224 to 1.76 eV for hybrid PCN-224(Zr/Ti). The Mott–Schottky plot indicates that the exchange of Ti produces a notable change in V_CB_, which enhances the charge transfer activity between the oxo-clusters and the H_2_TCPP linker. Adding Ti was found to intensely boost the production of ^•^O_2_^−^, which is the principal reactive species for the catalytic decay of MB dye. Therefore, the catalytic photodegradation performance of PCN-224(Zr/Ti) for MB dye improved after the inclusion of the photoactive metal Ti into the core of the Zr-MOF (Figure 41).

**Figure 42:**
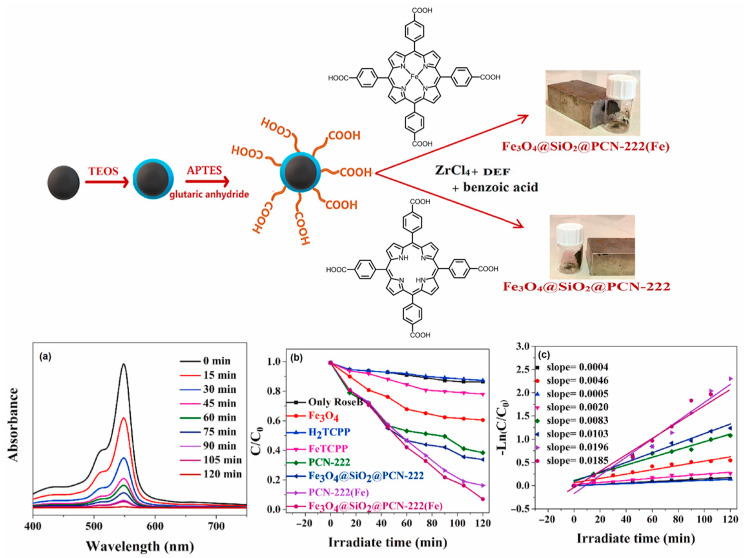
Synthesis of the Fe_3_O_4_@SiO_2_@PCN-222(Fe) composite. Solar light photodegradation of Rose Bengal dye in the presence of photocatalysts; (**a**) UV-vis spectra, (**b**,**c**) degradation kinetics. Adapted from Ref. [124].

Dehghanpour et al. developed a Zr-porphyrin-based magnetic photocatalyst, Fe_3_O_4_@SiO_2_@PCN-222(Fe) [124] (Figure 42).

The metalloporphyrin ligands present in the catalyst network act as anchors for absorbing the solar spectrum, transferring the excited electrons to the Zr_6_O_8_ clusters, and generating reactive oxygen species in an aqueous solution. The photogenerated reactive species such as e^−^, O_2_^•−^, and h^+^ produced on the surface of the catalyst enhance the degree of photodegradation of Rose Bengal dye (RoseB), RhB dye, and EryB dye compared to Fe_3_O_4_@SiO_2_ (Figure 42). Additionally, the separation of the photocatalyst is very easy owing to the magnetic properties of the catalyst. The photocatalyst can be smoothly isolated from the reaction mixture using a magnet.

**Figure 43:**
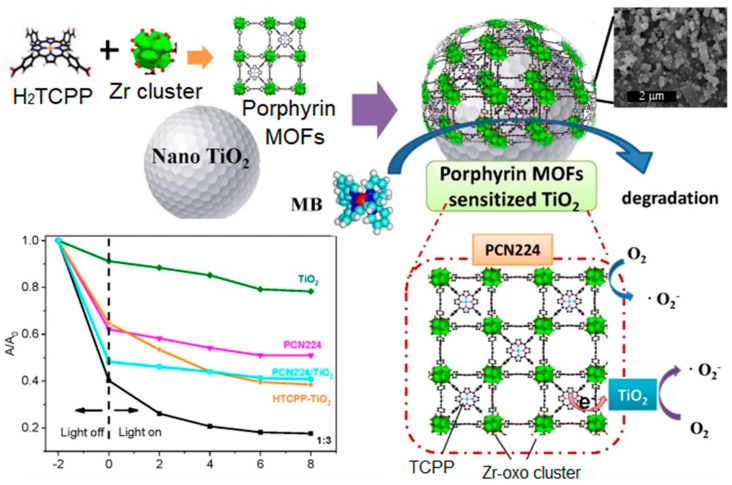
Fabrication of Zr-MOF-sensitized TiO_2_ (PCN-224@TiO_2_) composite. Catalytic degradation of MB dye in the presence of solar light and catalysts. Adapted from Ref. [125].

A Zr-porphyrin-sensitized TiO_2_ (PCN-224@TiO_2_) photocatalyst was used for the photodegradation of toxic contaminants [125]. The hybrid catalyst was fabricated in situ via the self-aggregation of porphyrin, ZrCl_4_, and TiO_2_ nanoparticles. PCN-224@TiO_2_ photocatalyst shows significant absorption in the frequency range of the solar spectrum. The separation of the photogenerated hole pairs significantly improves its photodegradation performance compared to the performances of PCN-224 or TiO_2_ alone. PCN-224@TiO_2_ (3:1) removed 93.2% of the MB dye under Xe lamp irradiation (Figure 43). Therefore, the fabrication of PCN-224 with TiO_2_ not only improves the photocatalytic performance but also increases the interaction between the active sites of the photocatalyst and dye molecules.

**Figure 44:**
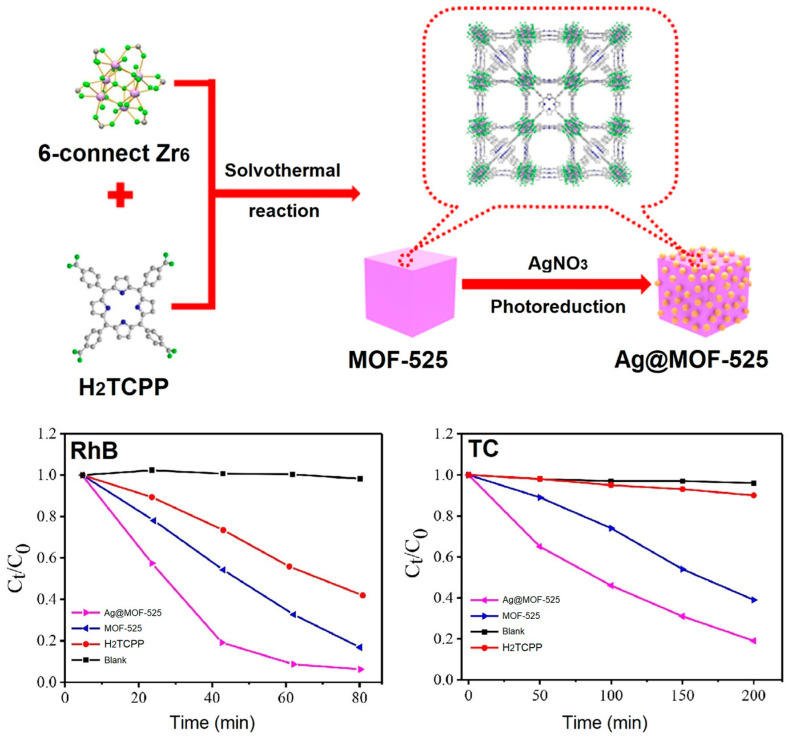
Synthesis of composite catalyst Ag@MOF-525 using the photoreduction process and its utilization in visible-light catalytic photodegradation of pollutants. Adapted from Ref. [126].

Recently, Yang et al. demonstrated a porphyrin-based hybrid catalyst (Ag@MOF-525) for visible-light catalytic photodegradation of pollutants in water [126]. Photoreduction of silver salts on the surface of MOF-525 under solar light irradiation was performed, which led to the formation of Ag@MOF-525. The incorporation of Ag plasmons onto the surface of MOF-525 not only boosts the light response range but also promotes electron transfer, thereby improving the separation of the photogenerated carriers. Under solar light irradiation, Ag@MOF-525 removed 91% of RhB within 60 min and 81% of tetracycline (TC) within 200 min. This removal efficiency is higher than that of MOF-525 and H_2_TCPP (Figure 44). Radical trapping analysis confirmed that h^+^ and O_2_^•−^ are the primary reactive species generated during the photodegradation process.

**Figure 45:**
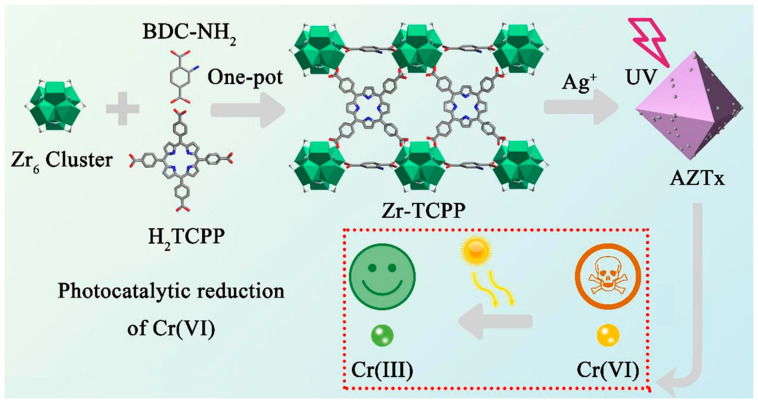
Fabrication of composite catalysts AZTx via photoreduction procedure and its utilization for the catalytic photodegradation of poisonous Cr(VI) ions in water. Adapted from Ref. [127].

Yang et al. also reported a porphyrin-containing composite photocatalyst (AZTx) for the visible-light catalytic reduction of poisonous Cr(VI) ions to the less poisonous Cr(III) in water [127] (Figure 45).

Zr-TCPP was prepared by a one-pot synthesis of the H_2_TCPP ligand and UiO-66-NH_2_. The incorporation of H_2_TCPP into Zr-TCPP improved its light-harvesting properties. Furthermore, the fabrication of Ag NPs on the surface of Zr-TCPP under ultraviolet light irradiation led to the formation of AZTx (Ag NPs@Zr-TCPP). The introduction of Ag NPs on the surface of Zr-TCPP enhanced the visible-light photoreduction efficiency of Cr(VI) ions in water. Under visible-light irradiation, the hybrid composite AZT5 (5 wt% ratio of Ag NPs to Zr-TCPP) reduced 94.1% of the highly poisonous Cr(VI) ions into less poisonous Cr(III) within 30 min under acidic conditions (Figure 45). The first-order degradation rate constant was approximately 3.6–5.4 times higher compared to that of Zr-TCPP. Therefore, the incorporation of Ag plasmons on the surface of Zr-TCPP increases the light-harvesting properties and facilitates the charge separation of the photogenerated charged species.

**Figure 46:**
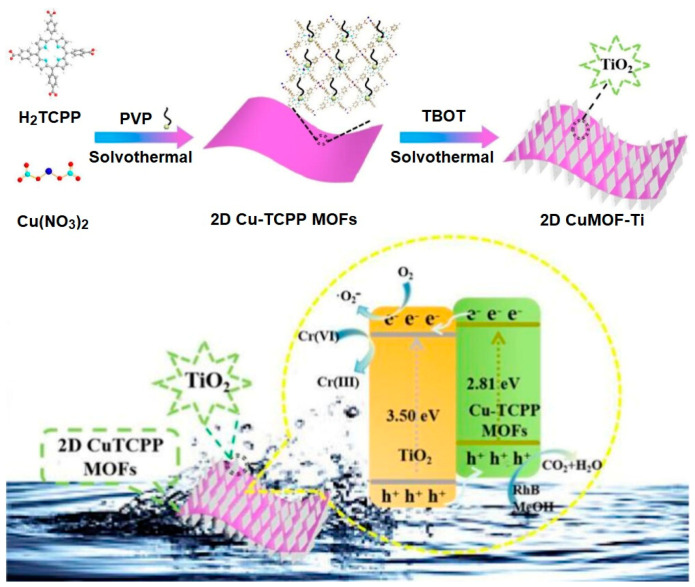
Construction of hybrid photocatalyst (2D CuMOF-Ti) and its utilization for the catalytic photodegradation of RhB dye and photocatalytic reduction of poisonous Cr(VI) ions in water. Adapted from Ref. [128].

A 2D Cu(II)porphyrin-based composite photocatalyst (2D CuMOF-Ti) was developed to eliminate pollutant dyes and reduce Cr(VI) under sunlight [128]. The hybrid catalyst was fabricated in two steps. In step one, 2D Cu-TCPP MOFs were constructed by the solvothermal reaction of H_2_TCPP and Cu(NO_3_)_2_ in the presence of the surfactant polyvinylpyrrolidone (PVP). In the second step, the reaction of the 2D Cu-TCPP MOFs with *n*-butyl titanate (TBOT) in DMF/ethanol led to the formation of 2D CuMOF-Ti (Figure 46). This hybrid composite shows a high absorption coefficient owing to the 2D ultrathin nanosheet morphology of the Cu-TCPP MOFs. The incorporation of amorphous TiO_2_ on the surface of the 2D nanosheets of Cu-TCPP MOFs not only increases the wider visible-light absorption but also enhances the charge separation and improves the photocatalytic performance. This composite showed solar light photodegradation of RhB dye, and its rate constant is 2.8 and 12.6 times higher than the rate constants of TiO_2_ and 2D Cu-TCPP MOFs, respectively. Additionally, this composite exhibits significant catalytic photoreduction activity for the conversion of toxic Cr(VI) to Cr(III).

**Figure 47:**
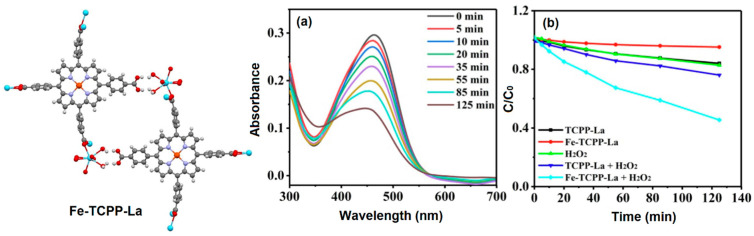
Fabrication of bimetallic photocatalyst Fe-TCPP-La for catalytic photodegradation of MO dye in water. (**a**) UV-Vis spectral change and (**b**) reaction kinetics. Adapted from Ref. [129].

Lan et al. reported a bimetallic metal–organic framework material for the photocatalytic degradation of methyl orange dye under visible-light irradiation [129]. The solvothermal reaction of Fe-TCPP (Fe-tetra(4-carboxyphenyl)porphyrin) with La(NO_3_)_3_·6H_2_O in the presence of a catalytic amount of hydrochloric acid in DMF leads to the formation of the bimetallic porphyrin-based metal–organic framework Fe-TCPP-La. In the crystal structure of Fe-TCPP-La, La^III^ acquires an eight-coordinate geometry (six oxygen atoms from the carboxylate groups of Fe-TCPP and two oxygen atoms from two water molecules), and the iron atom of the Fe^II^ ion in the metalloporphyrin presents a square-plane tetra-coordinated geometry. The Fe-TCPP-La MOFs have 3D porous topologies with large 1D channels and high porosity (total solvent area volume of 1740 Å^3^). Fe-TCPP-La exhibits good chemical and thermal stability and outstanding catalytic performance in the degradation of an aqueous solution of methyl orange dye. Superoxide radical anion (O_2_^•−^) and hydroxyl radicals (^•^OH) were the principal reactive species in the photodegradation of MO dye in the presence of solar light. Interestingly, in the presence of H_2_O_2_ as a cocatalyst, Fe-TCPP-La showed enhanced photocatalytic degradation of MO (Figure 47).

## 5. Conclusions

To summarize, this review reports the remarkable progress in the development of porphyrin-containing metal–organic framework materials for the photocatalytic degradation of toxic pollutants from wastewater under visible-light irradiation. First, it starts with the discussion of the synthesis procedures for the P-MOFs. Several reliable methods, such as hydrothermal, solvothermal, microwave-assisted, sonochemical, mechanochemical, electrochemical, reverse microemulsion, slow evaporation, and vapor diffusion methods, have been used for the construction of MOFs. Solvothermal synthesis is widely used for constructing P-MOFs. Other methods have aimed to minimize the high energy consumption, which is expected to become the trend in the coming days. Various functional porphyrin MOFs have been fabricated by reacting porphyrin derivatives with metal ions or clusters. Two types of square planar porphyrin building blocks, H_2_TPyP and H_2_TCPP, have been used for the synthesis of the P-MOFs. These two porphyrin ligands provide two unique metal-binding sites (within the porphyrin core and the pyridyl or carboxy ligands). Other porphyrin-based bridging ligands have also been used to fabricate porphyrin-based MOFs. The current progress in the utilization of P-MOFs for the photocatalytic removal of toxic pollutants was systematically evaluated. The synthetic versatility of various porphyrins with metal clusters or ions allows the organization of porphyrin-based MOFs that can significantly benefit from improved light-harvesting properties and a variety of morphologies, topologies, and micropore sizes. The distinctive structure and variable functionalization of porphyrin-based MOFs increase the photocurrent response, enhance the charge transfer operation, and facilitate the photocatalytic decay of toxic chemicals. P-MOF materials absorb solar energy from solar light, and the electrons in the VB transfer to the CB, creating electron–hole pairs. These photogenerated hole–electron pairs participate in the photodegradation reaction to produce reactive oxygen species and degrade toxic contaminants into non-toxic CO_2_ and H_2_O. Intense electronic delocalization of the photoinduced charged species occurs on the surfaces of the P-MOFs. This delays the recombination of the photogenerated species, thus increasing their photocatalytic ability. The permanent porosity inside the cavities of P-MOFs provides the space required for the encapsulation of substrate molecules. In addition, large porous micropores provide a large number of active sites that interact with a large number of cationic, anionic, and neutral species. These robust P-MOFs not only harvest photons but also prevent the decomposition of reactive species during the degradation process and increase recyclability. Although consolidated procedures have been explored for the formation of various P-MOFs in the solid state, the catalytic photodegradation performance of the as-prepared P-MOFs is still restricted by the rapid recombination of photogenerated carriers, insufficient chemical stability, and low degradation efficiency. Moreover, the low yield and high cost of porphyrinic ligands restrict their industrial-scale use. Therefore, a discrete and favorable trend in this field is the design of composite materials in which P-MOFs are combined with several photofunctional nanomaterials to achieve synergistic effects. The aim of this type of fabrication is to upgrade the photodegradation capacity in terms of light-harvesting ability, significant charge separation, large surface area, and sufficient chemical photostability, separation, and recovery from aqueous media, which are crucial for industrial applications. The application possibility of MOFs based on several porphyrin derivatives is expected to increase noticeably in the future with greater improvements in this class of materials. Further exploration is required for the removal of hazardous pollutants from P-MOFs, which should address the following issues:

1. Although the synthetic procedures for P-MOFs are simple, their costs remain high. Researchers must explore cost-effective procedures for the construction of P-MOFs.
2. Currently, experiments on the degradation of toxic contaminants using P-MOFs are conducted at the laboratory scale. In practice, other compounds can perturb the degradation reactions. Therefore, these specific experiments must be analyzed and optimized for industrial-scale use.
3. The structural durability under humidity and temperature of P-MOFs affects their degradation efficiency. Still, the majority of P-MOFs shows a high susceptibility to moisture, leading to a collapse of their geometrical architectures when exposed to aqueous mediums. Therefore, the improvement of water stability in P-MOFs has become a crucial area of investigation for scientists.
4. Infrared light constitutes 50% of the solar spectrum. Therefore, the fabrication of P-MOFs should be performed in such a way that renders them more appropriate for the utilization of infrared light.

In conclusion, this review article described the trends in the design and fabrication of porphyrin-based metal–organic framework photocatalysts. The distinctive and flexible functionality of porphyrins, in combination with metal ions or nodes, contributes to the formation of porphyrin-based MOFs with high porosity, which can be used as an ideal platform for the fabrication of various materials with precise properties and tailorable structures. This article demonstrated that porphyrin-containing metal–organic compounds constructed from porphyrin ligands and metal ions or clusters can be perfect photocatalysts for collecting solar light from a wide frequency range of the visible-light spectrum. These materials exhibit significant catalytic photodegradation of hazardous pollutants in wastewater under solar irradiation. This is because of their customizable crystalline structures, large number of metallic active sites, high porosity, and good photosensitivity. High solar-light harvesting in the visible region and significant electronic delocalization over the conjugated porphyrin rings make them more attractive than metal oxide nanoparticles. Versatile P-MOFs in conjugation can incorporate several semiconducting materials to fabricate composite materials with improved photocatalytic activities. Therefore, the process of wastewater purification is expected to greatly benefit from these P-MOFs, and future research is expected to reveal their new and important properties as well as avenues for their utilization in energy-related fields. With the joint efforts of material chemistry researchers, porous P-MOFs can achieve large-scale industrial production in the near future. The choice of metal ion (both for metallated-porphyrin or linker) is also crucial for the fabrication of P-MOFs. By altering the metal active centers, P-MOFs can gain control over the crystalline structures (morphology and pore size), specific area, metallic active sites, band gap energy, improved photocurrent response, enhanced charge transfer capability, strengthened conductivity, and improved photocatalytic reducibility. We believe that this review article can provide practical information for material chemistry researchers and serve as a reference for the use of P-MOFs for the degradation of toxic contaminants in wastewater.

## Data Availability

Not applicable.

## Nomenclature

AOP	Advanced oxidation process
AM	Amaranth
AO	Acid orange
BET	Brunauer–Emmett–Teller
BCG	Bromo cresol green
CuTPyP	[5,10,15,20-tetrakis(4-pyridyl)porphyrinato]copper(II)
CB	Conduction band
DMF	*N*, *N*-dimethylformamide
DMA	*N*,*N*′-dimethylacetamide
EBT	Eriochrome Black T
Eg	Band gap energy
H_2_TCPP	5,10,15,20-Tetrakis(4-carboxyphenyl) porphyrin
H_2_TPyP	5,10,15,20-Tetrakis(4-pyridyl)porphyrin
H_4_TCPB	1,2,4,5-tetrakis(4-carboxyphenyl)benzene
H_2_DPBPP	5,15-dipyridyl-10,20-bis(pentafluorophenyl)porphyrin
H_2_DCPP	5,15-di(4-carboxyphenyl)-10,20-diphenylporphyrin
MB	Methylene blue
MOF	Metal–organic framework
MO	Methyl orange
PdTPyP	[5,10,15,20-tetrakis(4-pyridyl)porphyrinato]palladium(II)
P-MOF	Porphyrin-based metal–organic framework
RhB	Rhodamine B
ROS	Reactive oxygen species
VB	Valence band
